# Neutrophil extracellular traps and DNases orchestrate formation of peritoneal adhesions

**DOI:** 10.1016/j.isci.2023.108289

**Published:** 2023-10-27

**Authors:** Julia Elrod, Annika Heuer, Jasmin Knopf, Janina Schoen, Lavinia Schönfeld, Magdalena Trochimiuk, Carolin Stiel, Birgit Appl, Laia Pagerols Raluy, Ceren Saygi, Leticija Zlatar, Sami Hosari, Dmytro Royzman, Thomas H. Winkler, Günter Lochnit, Moritz Leppkes, Robert Grützmann, Georg Schett, Christian Tomuschat, Konrad Reinshagen, Martin Herrmann, Tobias A. Fuchs, Michael Boettcher

**Affiliations:** 1 Department of Pediatric Surgery, University Medical Center Hamburg-Eppendorf, Hamburg, Germany; 2 Department of Pediatric Surgery, University Medical Center Mannheim, University of Heidelberg, Mannheim, Germany; 3 Department of Trauma, Hand and Reconstructive Surgery, University Medical Center Hamburg-Eppendorf, Hamburg, Germany; 4 Department of Internal Medicine 3 - Rheumatology and Immunology, Friedrich-Alexander-Universität Erlangen-Nürnberg (FAU) and Universitätsklinikum Erlangen, Erlangen, Germany; 5 Deutsches Zentrum für Immuntherapie (DZI), Friedrich-Alexander-Universität Erlangen-Nürnberg and Universitätsklinikum Erlangen, Erlangen, Germany; 6 Bioinformatics Facility, University Medical Center Hamburg-Eppendorf, Hamburg, Germany; 7 Department of Surgery, Universitätsklinikum Erlangen, Erlangen, Germany; 8 Division of Genetics, Department of Biology, Nikolaus-Fiebiger-Center of Molecular Medicine, Friedrich-Alexander University Erlangen-Nürnberg, Erlangen, Germany; 9 Protein Analytics, Institute of Biochemistry, Faculty of Medicine, Justus Liebig University Giessen, Friedrichstrasse 24, Giessen, Germany; 10 Department of Internal Medicine 1 - Gastroenterologie, Pneumologie und Endokrinologie, Friedrich-Alexander-Universität Erlangen-Nürnberg (FAU) and Universitätsklinikum Erlangen, Erlangen, Germany

**Keywords:** Health sciences, Biological sciences, Immunology, Cell biology

## Abstract

Peritoneal adhesions are poorly understood but highly prevalent conditions that can cause intestinal obstruction and pelvic pain requiring surgery. While there is consensus that stress-induced inflammation triggers peritoneal adhesions, the molecular processes of their formation still remain elusive. We performed murine models and analyzed human samples to monitor the formation of adhesions and the treatment with DNases. Various molecular analyses were used to evaluate the adhesions. The experimental peritoneal adhesions of the murine models and biopsy material from humans are largely based on neutrophil extracellular traps (NETs). Treatment with DNASE1 (Dornase alfa) and the human DNASE1L3 analog (NTR-10), significantly reduced peritoneal adhesions in experimental models.

We conclude that NETs serve as essential scaffold for the formation of adhesions; DNases interfere with this process. Herein, we show that therapeutic application of DNases can be employed to prevent the formation of murine peritoneal adhesions. If this can be translated into the human situation requires clinical studies.

## Highlights

• NETs play a key role in peritoneal adhesion formation
• DNases offer a promising therapy to prevent adhesions
• Human adhesions contain NET-associated proteins
• DNase treatment doesn't negatively impact wound healing

## Graphical abstract

**Figure:**
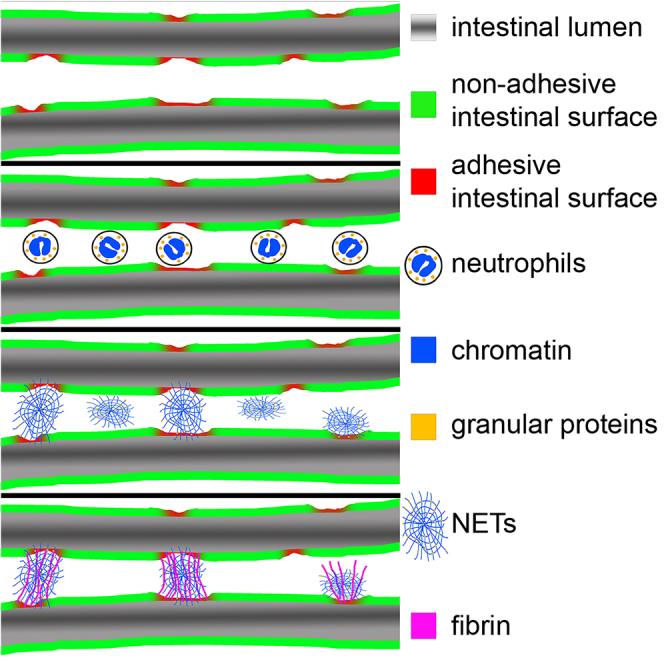

## Introduction

Peritoneal adhesions, a common consequence of serosal repair after abdominal interventions, represent a major burden for patients and surgeons alike. In fact, the formation of adhesions has been shown to occur as often as in 93–100% of patients following abdominal surgery and can cause serious complications such as intestinal obstruction, pelvic pain, and infertility.^1^ As a result, the quality of life of millions of patients throughout the world is affected by peritoneal adhesions. These lesions are also associated with considerable costs of roughly two billion US dollar per year in the US. Their actual burden in the medical setting is highlighted by a Scottish survey performed over a 10-year period. They suggested that 5.5% of all hospital re-admissions can directly be attributed to the formation of adhesions.^2^ Lastly, the formation of adhesions has not only been linked to a reduced quality of life and significant health care costs, but also mortality rates of 6–15%.^3^

Despite the clinical impact of adhesions, the pathomechanism of their formation is poorly understood. Peritoneal healing is a highly complex process involving hemostasis, inflammation, angiogenesis, formation of granulation tissue, deposition of extracellular matrix (ECM), and tissue remodeling.^4^ However, there is evidence that the most important element of peritoneal healing and the formation of adhesions after peritoneal injury during surgery is inflammation. After a peritoneal injury, the innate immune system reacts within hours through a plethora of mechanisms in order to clear pathogens and repair damaged tissue. These remain activated for several days and create a delicate balance which, if not successfully resolved, can tip over from protecting the host from microbes to mediating hyperinflammation, inhibiting healing, and increasing mortality.^5^

Neutrophils, the predominant leukocytes of acute inflammatory reactions, are immediately recruited into injured areas and remain the dominant cell population for about two days.^6^ They are (1) the most abundant immune cells in the human circulation, (2) regarded as the first line of defense of the innate immune system, and (3) the main leukocyte subset involved in the early phases of wound healing.^7^ In response to infection and/or injury, neutrophils form neutrophil extracellular traps (NETs), which consist of high molecular weight double-stranded DNA filaments that build robust scaffolds. These are decorated with histones and cytotoxic proteins, such as myeloperoxidase (MPO) and neutrophil elastase (NE), accounting for 70% and 20% of all proteins of NETs, respectively.^8^ Neutrophils release NETs by multiple mechanisms: (1) NETosis, a programmed cell death pathway, (2) non-lytic discharge of parts or their entire nucleus, and (3) mitochondrial DNA release, providing an additional DNA source for NET formation.^9^

NETs are “double-edged swords” as they regulate homeostatic and pathological inflammation. During infection NETs exhibit antimicrobial functions, trap and kill extracellular pathogens in blood and tissue.^9^ However, NETs also form during sterile inflammation. NETs stimulate platelet adhesion and coagulation^10^ and the proteolytic activity of aggregated NETs traps histones^11^ and contributes to the resolution of inflammation.^12^

While the function of NETs is essential in combating infection and inflammatory responses, a spatial and temporal inappropriate production of NETs can have detrimental effects. In fact, it has been shown that NETs contribute to the pathology of several inflammatory conditions, such as autoimmune diseases, wound healing, sepsis, and ischemia reperfusion injury.^13^^,^^14^^,^^15^ A common denominator of these disorders is the involvement of NETs as mediators of thrombosis and hyperinflammation and of the occlusion of vessels and ducts.^16^^,^^17^^,^^18^^,^^19^^,^^20^

To date, no molecular therapies exist that interfere with the formation of peritoneal adhesions. Instead, therapeutic interventions to impede the formation of adhesions are limited to bioabsorbable films, placed on surgical wounds to prevent adhesions of the peritoneum and the viscera. A number of barrier agents with various features are also available. However, these measures are complicated and time consuming. Furthermore, a recently published large Cochrane review, including 19 randomized controlled trials on the prevention of adhesion after gynecological surgery, did not reveal conclusive evidence of the effectiveness of such approaches.^21^ Taken together, a treatment preventing peritoneal adhesions represents an unmet medical need.

Based on response-to-stress and inflammation as potential triggers for peritoneal adhesions, we hypothesized that NETs play an important role in the formation of such adhesions. Furthermore, we considered that therapeutic application of DNases that cleave NETs may represent a new treatment option for peritoneal adhesions.

## Results

### Kinetic of the formation of peritoneal adhesions

**Figure 1:**
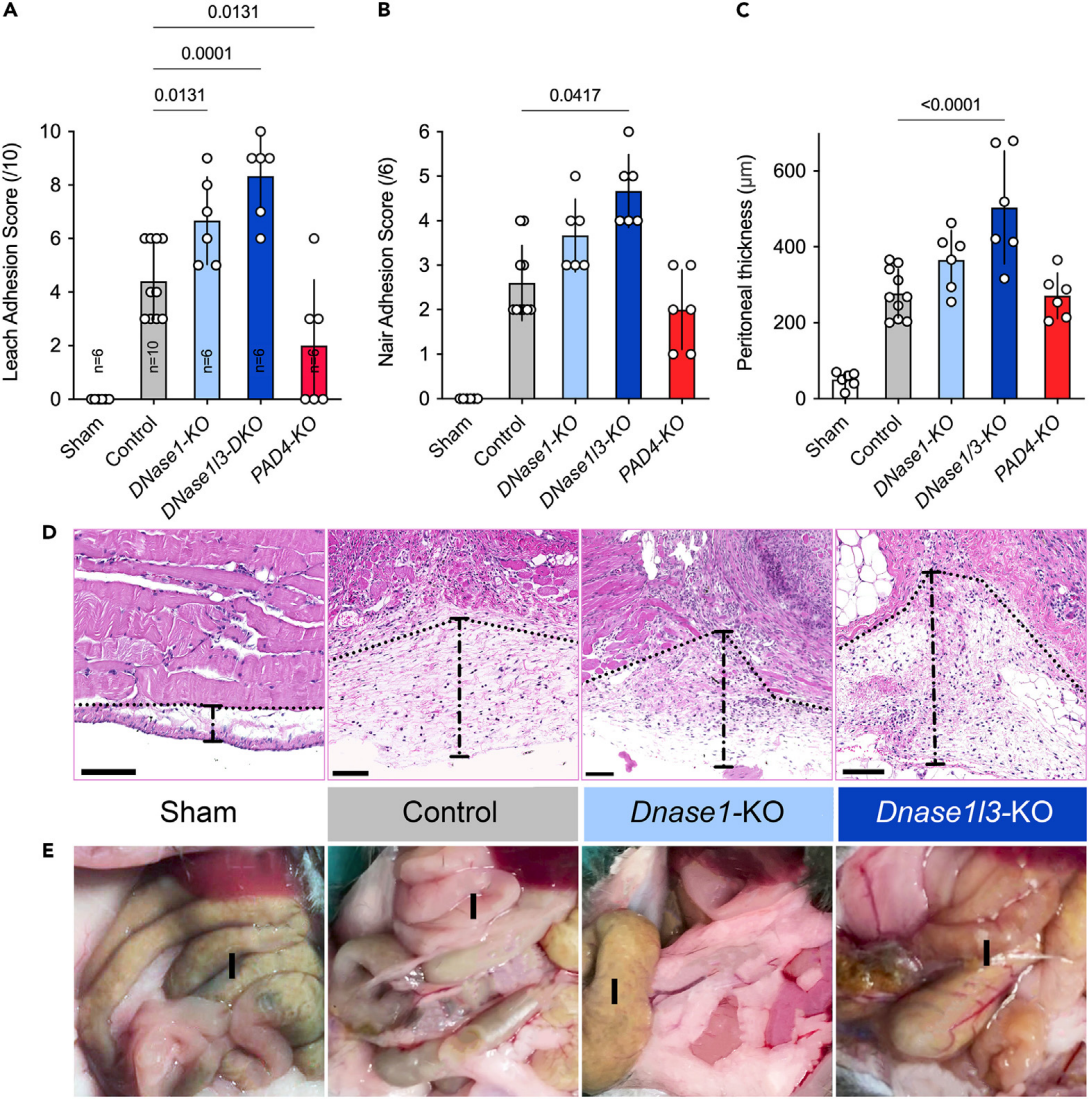
The absence of DNases precipitates the formation of adhesions (A and B) Animals with targeted deletion of *Dnase1* and even more *Dnase1l3* displayed significantly higher Leach and Nair adhesion scores than controls. In mice with PAD4-KO adhesions were reduced compared to controls (C) Adhesions are reflected by significantly increased thickness of the parietal peritoneum in the area of the peritoneal injury. (D) Representative HE images of DNases knockout mice compared to controls and shams. (E) Representative images of abdominal adhesions in the DNases knockout mice compared to controls and shams. I is marking the intestine. Data shown as mean ± SD. Statistics: ANOVA with Dunnett’s correction or Kruskal-Wallis test with Dunn’s correction.

We first determined the time course of extracellular DNA formation in the course of NET formation after injury (laparotomy) in wild-type mice (see STAR Methods section). We visualized extracellular DNA using SYTOX orange and observed a peak 72 h after the induction of adhesions (Figure S1). We observed the cumulative maximum of adhesions at 21 days using the Leach and Nair adhesion score (Figure 1). Therefore, we used both day 3 as well as day 21 post injury to assess the effects of DNases on NET formation and abdominal adhesions as shown in Figure S2.

### Laparotomy-induced adhesions in *Dnase1-*and *Dnase1l3-*KO mice

First, we examined the role of two endogenous DNases; DNASE1 and DNASE1L3 in abdominal adhesions. It is known that deficiency of the former contributes to lupus, whereas the latter is associated with scleroderma and autoimmunity.^22^^,^^23^^,^^24^^,^^25^ Mice deficient in DNASE1 or DNASE1L3 insufficiently metabolize extracellular DNA and nuclear remnants. We studied these knockout mice with the same protocol for the formation of adhesions. We observed that especially the knockout of DNASE1L3 and of DNASE1 to a lesser extent augmented formation of peritoneal adhesions (Figures 1A, 1B, and 1E). This finding underscored the importance of DNases in the regulation of abdominal adhesions. The most distinctive phenotype with respect to the formation of adhesions was *Dnase1l3*-KO mice, indicating that DNASE1L3 is of paramount importance to prevent the formation of adhesions. The peritoneal thickness was also increased in these DNase mutant mice with more pronounced results in DNASE1L3 KO than *Dnase1*-KO mice (Figures 1C and 1D). In mice with PAD4-KO, which prevents NETs from aggregating (aggNETs),^26^ adhesion formation was reduced (Figure 1A).

**Figure 2:**
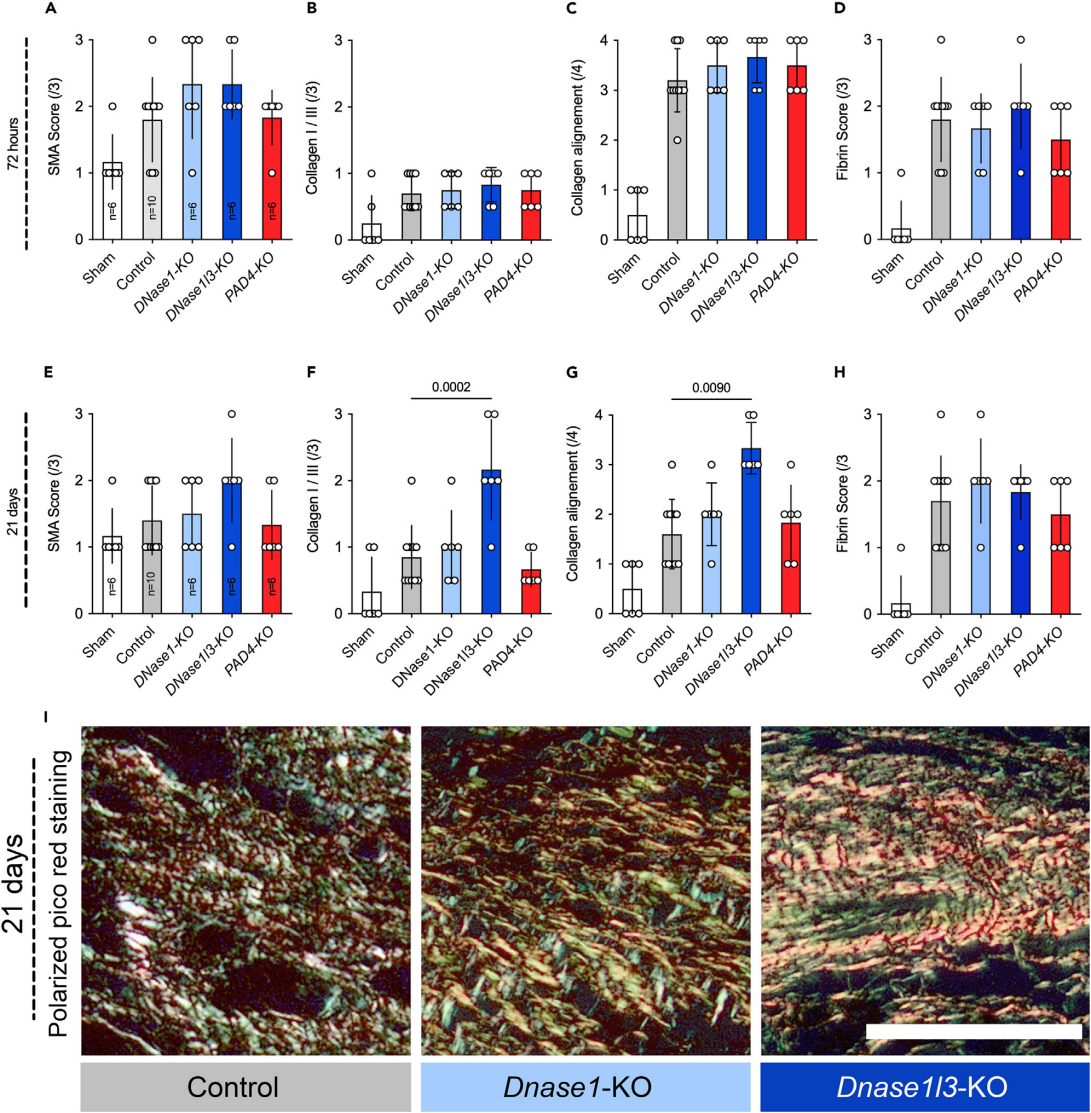
DNASE1L3 affects wound healing by inducing intensive collagen deposition (A and E) DNases appear not to affect SMA in short- and long-term which is a marker of wound contractility. (B, C, F, G, and I) Collagen 1:3 ratio and collagen alignment are used to access wound maturation. In *Dnase1l3* knockout mice very high levels of collagen I and III were to be found. Even after three weeks, the collagen fibrils remained aligned in parallel. This pattern is often associated with an immature wound and fibrosis. (D and H) Fibrin remained the same in all groups. Data shown as mean ± SD. Statistics: Kruskal-Wallis test with Dunn’s correction.

Upon closer examination, DNase treatment not only affected the formation of adhesions, but also influenced various aspects of peritoneal wound healing, as seen by an altered histology of the affected area (Figures 2A–2H). Compared to controls, the *Dnase1l3*-KO mice displayed a significantly higher collagen type I to type III ratio (Figures 2F and 2I) three weeks after injury. This adds further evidence to the role of DNASE1L3 in fibrosis.^22^ Other factors like fibrin or small muscle actin were not affected by the DNase treatment. The PAD4 knockout appears not to affect wound healing (Figures 2A, 2D, 2E, and 2H).

None of animals had complications after the laparotomy, such as wound infection or incisional hernia. As reported previously, PAD4-KO mice or mice that received DNases had improved laparotomy scars which as reported previously by our group.^14^

### Neutrophil- and NET-markers during laparotomy-induced adhesions formation

As DNases cleave NETs, we tested whether the absence of DNases induced peritoneal NET formation after abdominal injury. While the absence of DNases did not affect the total amount of Ly6G positive neutrophils (Figure S3A and S3G), *Dnase1l3*-KO mice showed significantly higher levels NETs formation, as measured by staining for myeloperoxidase (MPO), neutrophil elastase (NE) (Figure S3B, S3C, S3F, S3H, S3J), and citrullinated histone H3 (citH3) (Figure S3D, S3I, S3J). Moreover, control mice demonstrated very high levels of DNases (especially DNase1l3), which supports the hypothesis that DNases have a crucial role in the formation of peritoneal adhesions (Figure S3J). When measured NE activity in the surgical sites, we observed a robust NE activity (Figure S4A). Importantly, this activity was resistant to both the pharmacological (sivelestat) as well as the endogenous inhibitor α1-antitrpysin (α1-AT) (Figure S4B, S4C).

### Laparotomy-induced adhesions in WT mice-treatment with DNases

**Figure 3:**
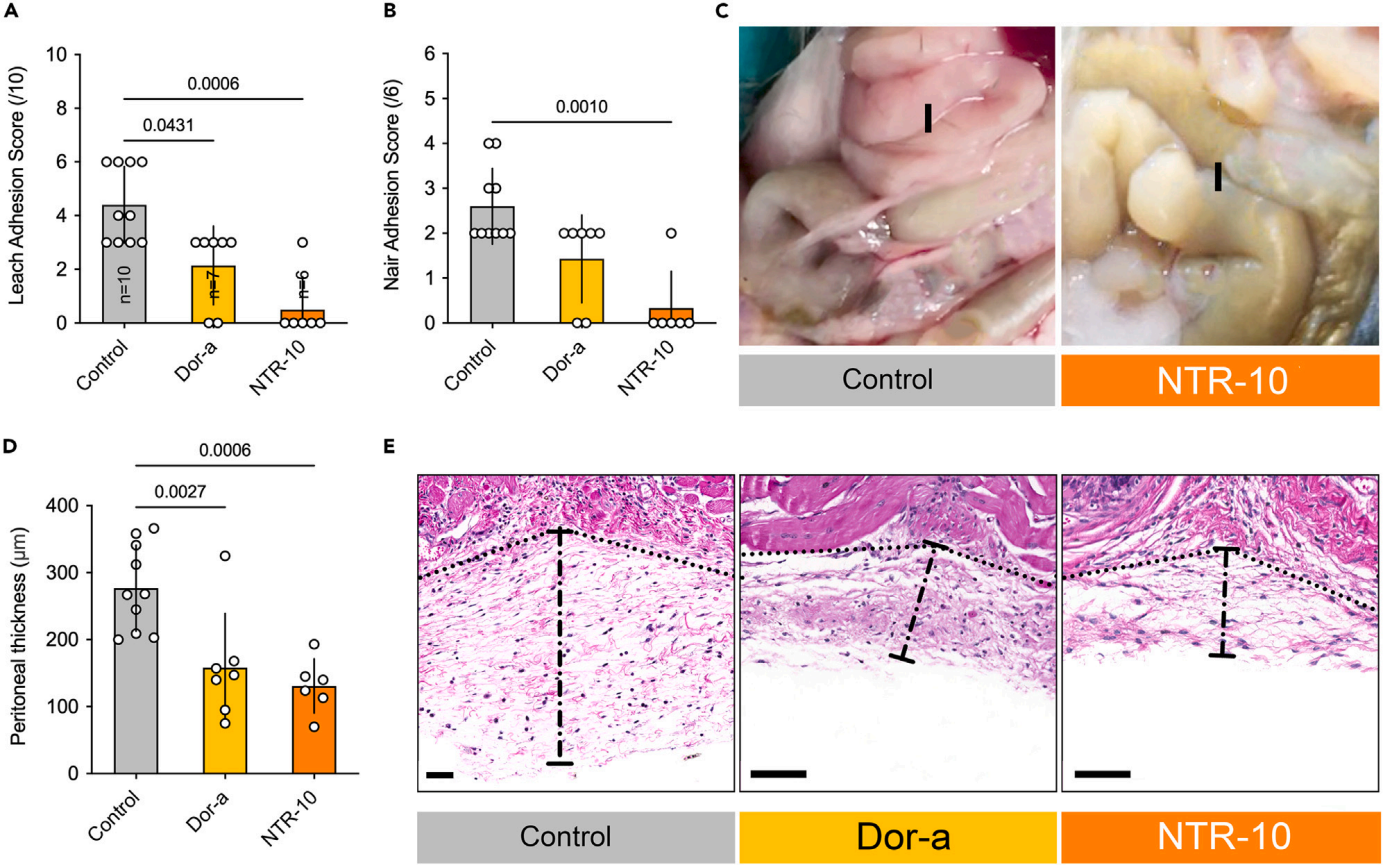
Topical NTR-10 significantly reduces the formation of adhesions *(*A‒C) The Leach and Near score were used to access the formation of adhesions at day 21. The most effective treatment option was NTR-10. (D and E) The thickness of the parietal peritoneum was significantly reduced in mice that received topical DNase treatment when compared to controls. (E) Representative images of the peritoneal thickness. I is marking the intestine. Data shown as mean ± SD. Statistics: ANOVA with Dunnett’s correction or Kruskal-Wallis test with Dunn’s correction.

We next aimed to identify the procedure that most effectively reduces and/or prevents the formation of peritoneal adhesions. Dornase alfa and/or NTR-10 were applied topically during the surgery and also systemically 24 h and 48 h afterward. NTR-10 significantly reduced adhesions at day 21 post-surgery; evaluated with the Leach and Nair adhesion scores (Figures 3A and 3B). Severity of adhesion was reflected by peritoneal thickness, which was significantly reduced by treatment with dornase alfa or NTR-10 compared to controls (Figures 3C–3E).

### Anastomosis and deserositation; treatment with DNASE1

**Figure 4:**
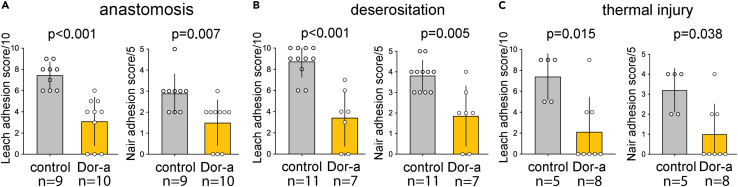
Dornase alfa reduces the formation of adhesions but maintains wound healing To test various typical clinical scenarios, mice were subjected to anastomosis of the small intestine (A) deserositation (B) and thermal injury (C) Treatment with dornase alfa prevented the formation of adhesions in all three scenarios almost completely. Additionally, it did not affect the rate of incision hernia, suture insufficiency, peritonitis, or mortality but rather improved it. Data shown as mean ± SD. Statistics: Kruskal-Wallis test with Dunn’s correction.

Since dornase alfa is commercially available, we continued the studies with dornase alfa. We evaluated the effects of dornase alfa on wound healing in two typical clinical settings: (1) intestinal anastomosis and (2) deserosation as a consequence of abdominal surgery. Assessing the effects of DNase on “beneficial” wound healing is of importance to surgeons, as DNases could potentially facilitate the spread of bacteria or delay wound healing. However, our results showed that mortality rates among animals treated with dornase alfa undergoing deserosation or intestinal anastomosis were not elevated in comparison to controls; i.e., animals treated with inactivated dornase alfa (failure of intestinal anastomosis: Dornase alfa 0.0% vs. controls 10.0%, p > 0.05; deserosation: Dornase alfa 22.2% vs. controls 29.3%, p > 0.05). No case of wound infection or incisional hernia was found. In contrast, peritoneal adhesions were again significantly reduced in mice treated with dornase alfa (Figures 4A–4C). These findings clearly demonstrate that application of DNases reduced the formation of adhesions without negatively affecting wound healing.

### Topical treatment with dornase alfa changes gene expression in peritoneal cells

Dornase alfa treatment ameliorated immune response and reduced leukocyte activation. mRNA analysis of murine peritoneal cavity cells showed that 2875 of the 3718 differentially expressed genes were downregulated after dornase alfa treatment (Figure S5A). The gene ontology (GO) terms regulation of leukocyte activation (Figure S5B) and activation of immune response (Figure S5C) were significantly enriched in the dornase alfa treated peritoneum. The top 20 most significantly up and downregulated genes were related to leukocyte adhesion (Figure S5B), activation of the immune response (Figure S5C), NET-associated genes (Figure S5D), and genes involved in nucleosome assembly (Figure S5E).^27^ The 5 most upregulated genes were Il31ra (IL31 receptor alfa), 493341K16Rik (K16RIK.), Bpifa2 (BPI Fold Containing Family A Member 2), Masp2 (Mannan-binding lectin-serine-protease 2), and Bpifbb1 (BPI Fold Containing Family B Member 1) The 5 most downregulated genes were Dmbt1 (Deleted In Malignant Brain Tumors 1), Epcam (Epithelial cell adhesion molecule), Wfdc2 (WAP four-disulfide core domain protein 2 or human epididymis protein 4), Aqp5 (Aquaporin 5), Clca3a2 (Chloride channel accessory 3A2). These are characterized by the GO GO:0002694 (regulation of leukocyte activation), GO:0002791 (regulation of peptide secretion), GO:0002253 (activation of immune response), GO:0019221 (cytokine-mediated signaling pathway), and GO:0002443 (leukocyte mediated immunity).

### Human adhesions contain neutrophil proteins and extracellular, NET-like DNA

**Figure 5:**
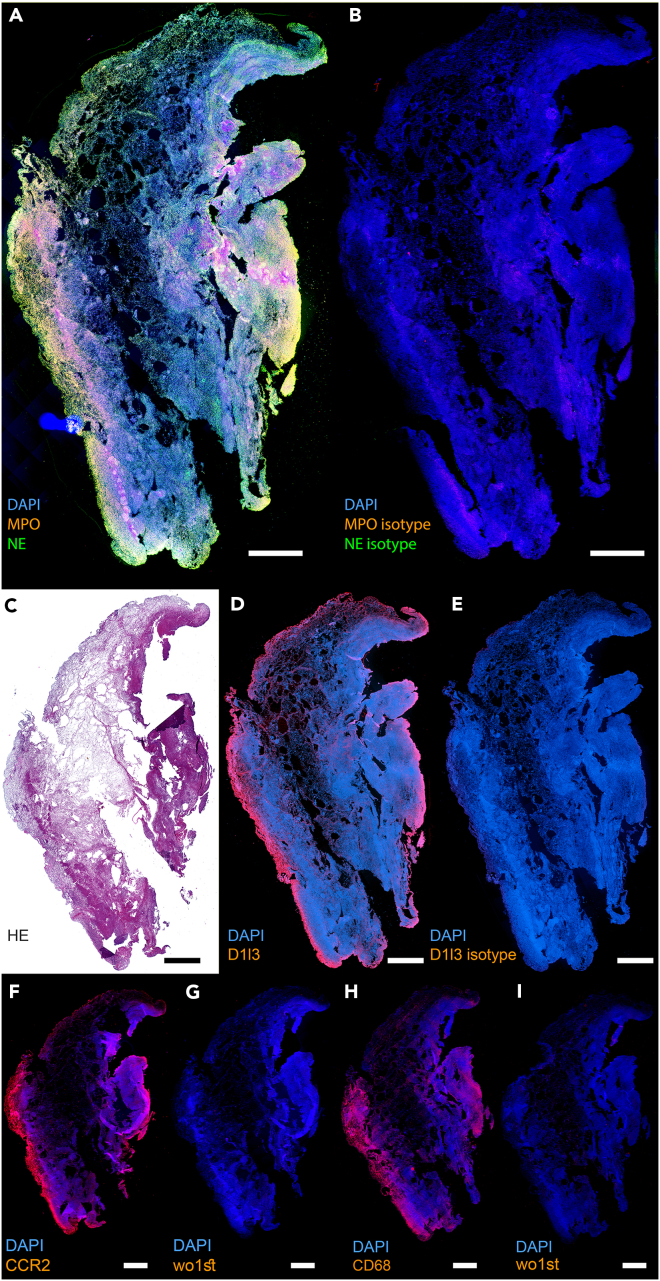
Human adhesions show NETs, DNase1l3, CCR2, and CD68 expression (A) Paraffin section of a human adhesion shows expression of the characteristic NET proteins myeloperoxidase (MPO, red) and neutrophil elastase (NE, green). (B) Isotype control for MPO and NE from (A). (C) HE staining of the same human adhesion. (D) Human adhesion also shows expression of DNase1l3 (red). (E) Isotype control for the staining of DNase1l3 in (D). (F and H) the monocyte marker CCR2 (red) and the macrophage marker CD68 (red) are also expressed in human adhesions. (G and I) control for the staining of CCR2 and CD68, respectively. (A,B,D,E, F, G, H, I) DNA was counterstained using DAPI. The size bar represents 100 μm.

To find out whether NETs are also components of human peritoneal adhesions, we analyzed human surgery material. As shown in representative images (Figure 5 and S6), the NET-associated proteins NE and MPO were expressed in the human peritoneal adhesions (Figure 5A). Additionally, the expression of DNASE1L3, especially at the margins of the adhesion, was detected (Figure 5D). Monocyte and macrophage associated peptides CCR2 and CD68 were also expressed in the marginal region of the adhesions (Figures 5F and 5H). The isotype controls (Figures 5G and 5I) were negative for the fluorescence signal, confirming the specificity of the signal obtained for NE, MPO, DNASE1L3, CCR2, and CD68. Figure 5C shows a hematoxylin and eosin (HE) staining of the same biopsy. Enlarged details of the surgical biopsy material are depicted in Figure S6 with additional positive immunofluorescence signals for the expression of the hallmark NET marker citH3.

To validate the presence of NET-borne proteins in human adhesions, we performed high-resolution mass spectrometry. The following NET-borne proteins were detected with high confidence: NE, MPO, Cathepsin G, S100-A8, and S100-A9. The secretory Proteinase 3 that is reportedly not incorporated in NETs was missing in all samples (Figure S7).^28^

### Adhesions contain modified fibrin

**Figure 6:**
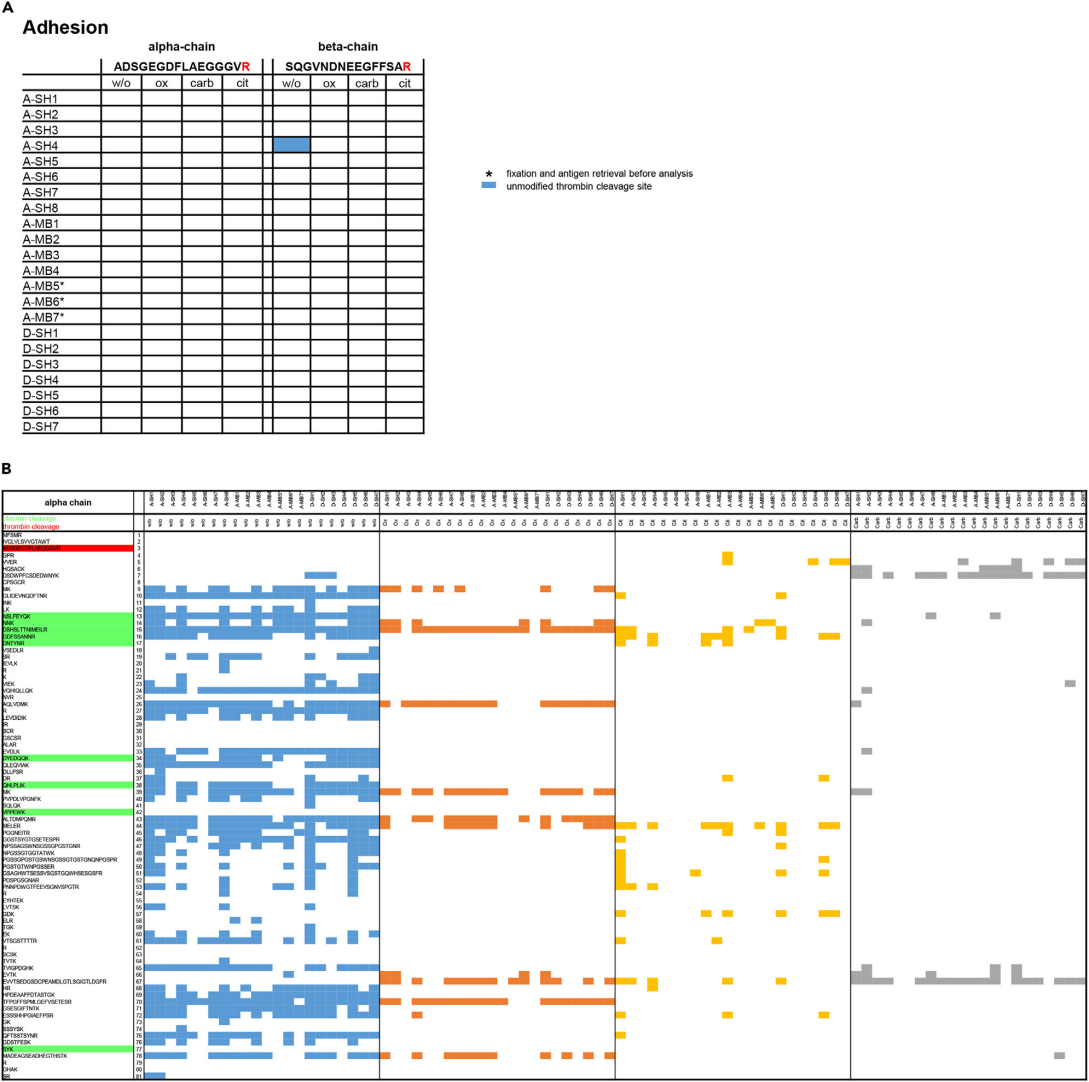
Human adhesions contain post-translationally modified fibrin High resolution mass spectrometry analysis with subsequent peptide and post-translational modification analysis was performed on surgical samples of human adhesions. (A) An unmodified peptide containing the thrombin cleavage site was only detected in one sample and corresponded to the thrombin cleavage site in the beta chain as indicated by the blue square. (B) Peptides identified corresponding to the fibrinogen beta chain in different samples of human adhesions. These peptides were either unmodified (w/o, blue box), oxidized (ox, orange box), citrullinated (cit, yellow box), or carbamylated (carb, gray box). The cleavage sites for thrombin are marked in red, and the major cleavage sites for plasmin in green. Note that the adhesions contain many peptides that had been oxidized, citrullinated, and carbamylated posttranscriptionally. Many of these are clustered near the plasmin cleavage sites. ∗ samples were fixed and subjected to antigen retrieval before mass spectrometry analyses. No major differences between fixed and unfixed samples were observed in the peptide analysis.

Interestingly, the NET markers anti-DNA, citH3, and MPO also co-localized with fibrin in surgical biopsy material (Figure S8). To evaluate the impact of neutrophils and NET-derived protein modifications of fibrin on the formation of adhesions, we performed high resolution mass spectrometry on human adhesions from abdominal surgery. The mass spectrometry data were subsequently analyzed for peptide sequences of alpha, beta, or gamma chains of fibrinogen with or without modification. We first examined the thrombin cleavage sites in the alpha and beta chains, since cleavage by thrombin catalyzes the formation of fibrin from fibrinogen. Strikingly, only a single unmodified peptide was found in the beta chain of one sample. No peptide sequences before the thrombin cleavage site, nor the site itself were found for the alpha chains and the beta chains of all other samples (Figure 6A). This indicates that the examined adhesions contained fully processed fibrin. When we analyzed white clots from drainage fluid of patients after abdominal surgery (n = 7), these contained a similar composition as the collected adhesions (Figure 6A).

We next investigated the region of the major cleavage sites for plasmin, which conducts fibrin clot degradation. The peptide containing the cleavage site, and peptide sequences surrounding these sites, were highly modified in all chains of all samples. Especially oxidized and citrullinated peptides were detected but also a few carbamylated ones (Figure 6B and Figure S9). Oxidation and citrullination are common markers of inflammation and can be mediated by neutrophils and NETs-borne enzymes, respectively.

Plasmin(ogen) displayed a lower Mascot score than a2-antiplasmin and a much lower than the fibrin chains. The plasminogen activators were virtually absent (Figure S10). This adds to the explanation for the long-lasting proteolytic resistance of adhesions. There were no obvious differences between adult and pediatric samples.

## Discussion

Our findings suggest that NETs and DNases play a pivotal role in the formation of peritoneal adhesions in mice and humans. We support previous research, which suggests that the pathogenesis of the formation of adhesions is based on a combination of inflammation, coagulation, and fibrinolysis.^29^

It is established that the inflammatory phase precedes wound healing. However, there are also controversial reports: (1) Wounds in areas with inherently lower levels of macrophages, neutrophils, and T cell infiltration, such as oral wounds, heal instantly with marginal inflammation and scar formation,^30^ (2) neutrophil depletion in mice accelerates the re-epithelialization rate of uninfected diabetic wounds,^31^ (3) in the wounds of diabetic mice elevated levels of citrullinated histone H3 (citH3) were found and healing was delayed; (4) wound healing was accelerated in peptidylarginine deiminase 4 (*PadI4*)-KO mice which have very limited NETs formation when compared with WT mice, and (5) DNASE-1 accelerated healing of incisional wounds in diabetic mice.^7^

In peritoneal wound healing, NETs appear to have similar disadvantageous effects. This is supported by our findings that at sites of peritoneal injury NETs were found and Dnases were expressed in both humans and mice. This finding suggests that NETs and Dnases regulate the formation of adhesions. Indeed, lack of DNASE1L3 resulted in massive deposits of collagen in peritoneum and increased formation of peritoneal adhesions. As such, it is also not surprising that a lack of DNASE1L3 has been associated with systemic sclerosis in previous studies.^32^

Assessment of peritoneal adhesions revealed widespread abundance of NETs characterized by extracellular DNA and NE activity. NETs may likely represent scaffolds for peritoneal adhesions. Thus, injury leads to recruitment of neutrophils which increase steadily at inflammatory foci, where they form NETs. The formation of NETs is further fostered by the high levels of HMGB1 in patients who underwent abdominal surgery.^33^ MGB1 reportedly promotes NET formation through engagement of the TLR4 receptor and therefore exacerbates damages in the ischemic brain or intestinal ischemia.^34^^,^^35^^,^^36^ The formation of NETs and the presence of monocytes in the marginal regions of human adhesions is in line with a recent publication by Tsai et al. showing that neutrophil recruitment and NET formation contribute to adhesion formation in a murine model of peritoneal adhesions.^37^ Contrary the findings in the murine model, we detected the presence of CD68-positive macrophages in the marginal regions of human adhesions. A distinct role for macrophages in iatrogenic procedures such as abdominal surgery in contrast to focal thermal or laser-induced peritoneal injures was reported by Zindel et al.^38^ In the setting of iatrogenic abdominal surgery macrophages may cause detrimental scaring while attempting to repair the wound and therefore fostering the formation of adhesions. Additionally, in homeostatic conditions, NETs are mainly degraded by DNases, provided for instance by macrophages.^39^ This gives another explanation for the presence of macrophages in the marginal regions of adhesions as detected in our samples. When NETs reach high local densities, they aggregate (aggNETs) and form structures that span over several centimeters that can easily bridge two intestinal loops. Since DNA builds the framework of these aggNETs, they are robust, sticky, highly flexible, and elastic. If they are not cleaved by DNases in time, they go through a certain degree of maturation, activate platelets and fibrinogen and are decorated with high molecular weight fibrin. The latter physically strengthen these structures by providing attachment points for fibroblasts, endothelial cells and, smooth muscle cells and immune cells, which eventually organize and develop into mature adhesions.

The study’s results are promising and DNase therapy to prevent the formation of adhesions is ready to be evaluated in a clinical study. As our data show, DNases do not appear to negatively affect wound healing. Indeed, our results suggest an enhanced physiological wound healing process after treatment with DNases, particularly with NTR-10. The main effect appears to be the reduction of NETs-associated inflammation. This is also reflected by the RNA seq data. We observed that DNases application or KO-Models reduced and increased the formation of adhesions, respectively; in both models wound healing was preserved. It did not enhance the incidence of hernia formation or anastomotic insufficiency.

The lysis of fibrin networks is temporally and spatially controlled. High flow of the surrounding fluid and high density of clot-borne plasminogen and its activators uPA and tPA supports fibrinolysis,^40^^,^^41^ whereas mechanical stretching as well as post-translational modifications of the binding sites for uPA and tPA and cleavage sites for plasmin stabilize the fibrin meshwork.^42^^,^^43^ The high mechanical stretching, the low fluid flow in the peritoneum and putatively the post-translational modifications all support the resistance of the adhesions to lysis. This explains why adhesions have prolonged long half-lives. During formation of canonical clots plasminogen as well as the plasminogen activators uPA and tPA are incorporated into the nascent clot in inactivated form.^44^ This prepares the clot for fibrinolysis as soon as uPA and tPA are activated.^45^ This usually occurs during clot formation and is executed by clot-associated plasmin.^46^

### Limitations of the study

While the findings of this study provide valuable insights into the role of NETs and DNases in peritoneal adhesion formation, several limitations should be acknowledged. To determine the safety and efficacy of DNase therapies in preventing peritoneal adhesions clinical trials are needed. Factors such as dosing, treatment duration, and potential side effects need thorough evaluation in human subjects. The study primarily focuses on the acute phase of adhesion formation. The long-term effects of NETs and DNase treatments on adhesion recurrence, patient outcomes, and potential complications remain to be explored.

In summary, we have shown that the formation of peritoneal adhesions is driven by the formation of NETs, and can be counteracted by DNase treatment. This poses an elegant treatment option, as DNases are cost effective and can metabolize extracellular DNA *in vitro.* Moreover, they are already being used for various other disorders, such as systemic lupus erythematosus and cystic fibrosis. So far, no serious adverse effects have been recorded with the application of DNases.^47^ In accordance, our data did not show any adverse effects of DNase treatment on wound healing.

## STAR★Methods

### Key resources table

**Table:** 

REAGENT or RESOURCE	SOURCE	IDENTIFIER
**Antibodies**		

Goat anti-mouse MPO (Figures 5, S3, S6, S8)	R&D Systems	RRID:AB_2250866
Rabbit anti-mouse NE (Figures 5, S3, S6)	Abcam	RRID:AB_1658868
Rabbit anti-mouse citH3 (Figures S3; S6; S8)	Abcam	CAT # ab219406
Rabbit anti-human DNase1L3 (Figures 5, S3, S6)	ThermoFisher	CAT# BS7653R
Mouse anti-DNA (Figure S8)	Merck	RRID:AB_93367
Rabbit anti-human fibrinogen (Figure S8)	Abcam	RRID:AB_10561758
Rabbit anti-human CCR2 (Figures 5, S6)	ThermoFisher	RRID:AB_11154101
Mouse anti-human CD68 (Figures 5, S6)	Abcam	RRID:AB_307338
Normal Goat IgG Control (Figures 5, S6)	R&D Systems	RRID:AB_354267
Rabbit IgG, polyclonal - Isotype Control (Figures 5, S6)	Abcam	RRID:AB_2631996
Rabbit IgG, monoclonal [EPR25A] Antibody (Figures 5, S6)	Abcam	RRID:AB_2687931

**Biological samples**		

Peritoneal tissue samples	Pediatric Surgery of the University Medical Center Hamburg-Eppendorf, Department of Surgery of the University Hospital Erlangen	N/A

**Chemicals, peptides, and recombinant proteins**		

Dornase alfa (Figure 4)	Roche	CAT# 0471672800
NTR-10 (Figure 3)	Neutrolis	N/A
Isoflurane	Baxter	N/A
Buprenorphine	Reckitt Benckiser	N/A
Target Retrieval Solution pH6	Agilent	CAT # S2369
Donkey Block	BioGenex	N/A
Fluoromount-G	Southern Biotech	CAT # 0100-01
Fluorogenic substrate MeOSuc-AAPV-AMC	Santa Cruz Biotechnology	CAT # sc-201163
SYTOX™ Orange Nucleic Acid Stain (Figure S1)	ThermoFisher	CAT # S11368
Neutrophil elastase from human leukocytes (Figure S4)	Sigma-Aldrich	CAT # E8140
Sivelestat sodium salt hydrate (Figure S4)	Sigma-Aldrich	CAT# S7198
Alpha-1-Antitrypsin (Figure S4)	Sigma-Aldrich	CAT# A9024

**Critical commercial assays**		

Picro Sirius Red Stain Kit (Figure 2)	Abcam	CAT# ab150681

**Deposited data**		

RNA data	Harvard Dataverse	https://doi.org/10.7910/DVN/V9NWVB

**Experimental models: Cell lines**		

JM8A3 embryonic stem cells (C57BL/6N origin)	University Medical Center Mannheim	RRID:CVCL_J959

**Experimental models: Organisms/strains**		

C57BL/6J *Mus musculus*	Jackson Laboratories	IMSR_JAX:000664
C57BL/6N *Mus musculus*	University Medical Center Mannheim	N/A
*DNase1*-KO *Mus musculus*	University Medical Center Mannheim	N/A
*Dnase1l3*-KO *Mus musculus*	University Medical Center Mannheim	N/A
B6.Cg-Padi4tm1.1Kmow/J *Mus musculus*	Jackson Laboratories	RRID:IMSR_JAX:030315

**Recombinant DNA**		

pX458	Addgene	RRID:Addgene_159654

### Resource availability

#### Lead contact

Michael Boettcher Michael.boettcher@medma.uni-heidelberg.de.

#### Materials availability

RNA data can be found at https://doi.org/10.7910/DVN/V9NWVB. All other data is included in the manuscript or supplements.

#### Data and code availability

RNA data can be found at https://doi.org/10.7910/DVN/V9NWVB. All other data is included in the manuscript or supplements. This paper does not report original code. Any additional information required to reanalyze the data reported in this paper is available from the lead contact upon request.

### Experimental model and study participant details

The study was approved by the Hamburg State Administration for animal research (73/15, 63/16). A total of 167 six–week-old female mice were utilized for the experimental model and all environmental parameters within the animal facility complied with the German guide for the care and use of laboratory animals (Animal Welfare Act)*.* The animals had a body weight around 18-19 g. All animals including the genetic knockouts (*DNase1*-KO, *Dnase1l3*-KO and PAD4-KO) used to examine the role of DNases in the process of the formation of adhesions and wound healing had the same genetic background (C57BL/6). The DNase1-KO and the *Dnase1l3*-KO mice were generated as described earlier.^12^^,^^48^^,^^49^ We obtained the WT as well as PAD4-KO mice from Jackson Laboratory and employed littermates for all treatment groups. Knockout animals were genotyped by Dr. Hermans-Borgmeyer, Center for Molecular Neurobiology UKE Hamburg.

Additionally, peritoneal samples of seven children and fifteen adults who underwent a second laparotomy within two weeks after their primary laparotomy were included in the study (Department of Pediatric Surgery of the University Medical Center Hamburg-Eppendorf from 2017 to 2019 and Department of Surgery of the University Hospital Erlangen from 2021 to 2022) and analyzed histologically. Samples were obtained only from cases with non-infectious conditions that lead to re-operative surgery. Anonymized tissue collection was in accordance with the guidelines of the medical research ethics committee of Hamburg (Ethik-Kommission der Ärztekammer Hamburg, PV5489) and with the 1964 Helsinki declaration and its later amendments. Written informed consent was obtained from the legal representatives.

#### Human sample collection and tissue sampling

Peritoneal tissue samples from the maximum of the adhesion were collected at the time of secondary relaparotomy surgery. Next, the peritoneal scar was dissected and evenly distributed into test tubes containing Bouin solution. White clots were harvested from the suction beginning immediately after surgery. Patients with infectious conditions were excluded from this current study. In total, we obtained adhesion samples from 29 patients, and analzyed them as described in “immunohistochemistry” and “immunofluorescence staining”. Morphologic images were captured using a 4K/12-megapixel camera (Panasonic LUMIX, Japan).

#### Generation of Dnase1 knockout mice

Dnase1 mutant mice were generated by CRISPR/Cas9 mediated mutagenesis in JM8A3 embryonic stem (ES) cells from C57BL/6N origin.^48^ In brief, ES cells were transfected with pX458 (obtained from Addgene, Watertown, MA, in which the Dnase1 specific gRNA-sequence 5' TGACATCGCTGTTATCCAAG 3' was inserted.^49^ GFP-expressing ES cells were sorted and mutations in individual ES cell clones were analyzed by sequencing the amplicon generated by primers flanking the target sequence in exon 3. One clone showing a 65 bp deletion from intron 2 into exon 3 was selected for blastocyst injections, generation of chimeric mice and further breeding with C57BL/6N mice. The deletion does not allow splicing into exon 3, and potential alternative splicing into exons 4, 5 or 6 containing the active sites for Dnase 1 enzymatic activity lead to frameshift mutations and premature stop codons.

#### Animal procedures

Mice were randomized into groups of equal size. For better standardization, a single surgeon performed all operations. Anesthesia was induced with 5% isoflurane (Baxter, Unterschleißheim, Germany) and maintained with 2.5% isoflurane gas delivered through a facemask. Preoperative antisepsis was performed with betaisadonna and all mice received 0.02 mg/kg bodyweight (BW) buprenorphine (Reckitt Benckiser, Mannheim, Germany) subcutaneously, 30 min preoperatively for analgesia.

The study involved two models: (1) adhesion model to assess the effect and implications of NETs and DNase treatment on the formation peritoneal adhesion.

#### Model 1: adhesion formation

Adhesions were induced using a bipolar electrocoagulation method^50^: Standardized lesions were inflicted on an area measuring 0.5 cm × 1.5 cm by sweeping the bipolar electrocoagulation forceps over the abdominal peritoneum for 2 seconds. The current was delivered using the following settings: Bipolar Soft, Effect 4, 40 Watts. The defects were subsequently closed using two interrupted sutures (6/0 Vicryl, Ethicon, Norderstedt, Germany) to induce an ischemic field around the traumatized area. The sutures were placed equidistantly (5 mm) along the defect and 1 mm from the wound’s edge.

To establish the most effective therapy, several treatment combinations using DNASE1 (Dornase alfa, Roche, Mannheim, Germany) with a dosage of 10 mg/kg BW, as well as NTR-10 (Neutrolis, Boston, USA), with a dosage of 1mg/kg BW were tested. The control groups received inactivated DNase1. DNASE1 or NTR-10 or inactivated DNase1 was applied with a volume of 0,5ml directly on the peritoneal lesion. Directly after application the abdomen was closed to ensure that the entire volume remained within the abdomen. The half-life of DNases is around two hours. Ultimately, a sham group without (1) the intervention, other than the laparotomy, and without (2) treatment was included.

To determine the effects of DNases on wound healing, three typical clinical scenarios were reproduced: (1) Deserositation – induced by rubbing a mini-prep on the wall of the small intestine, (2) Intestinal anastomosis - performed with a 8x0 Vicryl continuous suture after dissection of a small segment of the small intestine, and (3) Thermal injury - induced by heat exposure on the intestine using a red lamp with a distance of 1 meter for 10 minutes. In all mice the abdomen was closed using a single-layer continuous suture (Prolene 5-0; Ethicon, Norderstedt, Germany).

At the two timepoints (72 hours or 21 days), animals were euthanized after anesthesia using isoflurane as described above. Re-laparotomy was conducted and assessment of the formation of adhesions was performed. In all animals the adhesion between the peritoneal injury site and the small intestine was analyzed.

### Method details

#### Assessment of the adhesions

All adhesions were evaluated immediately after re-laparotomy. Macroscopic grading of the formation of adhesions was assessed by two independent surgeons, blinded to the animal groups and blinded to each other, using the Leach grade, as well as the Nair grade. The Leach grade was originally designed to score adhesions of the uterine horn and was thus modified for this study to evaluate peritoneal adhesions. The Leach score consists of three factors^51^: (1) severity of adhesions (0=no adhesion, 1=filmy avascular, 2= vascular or opaque, 3=cohesive attachment), (2) degree of adhesions (0=no adhesion, 1=adhesion separable with gentle traction, 2=adhesion separable with moderate traction, 3=requiring sharp dissection), and (3) extent of adhesions (0=no adhesion, 1=1-25%, 2=26-50%, 3=51-75%, 4=76-100%).

The Near score consist of two factors^52^: (1) macroscopic adhesions (0=no adhesion, 1=single band of adhesion between viscera to abdominal wall, 2=two bands between viscera to abdominal wall, 3=more than two bands to abdominal wall), and (2) microscopic adhesions (0=no fibrosis, 1=fibrosis with thin collagen bundle, 2=tissue with wider and less vascularized collagen fibrosis, 3=tissue with thick collagen bundle).

#### Microscopic grading

All specimens were evaluated histologically. All specimen were washed in phosphate buffered saline (PBS) and fixed in 10% buffered formalin before being embedded in paraffin and cut into 3μm thick sections, slides were then stained using hematoxylin and eosin (HE) and examined by two researchers who were blinded to the groups in light microscopy, using a magnification of ×4 and x10. Assessment of wound healing (epithelialization) was carried out in a standardized manner and expressed as a percentage of the whole wounded area. The unhealed wound was measured as the distance between both edges of the wound and the total wound diameter as the distance between the wound edges.

#### Immunohistochemistry (HE, Ly6g, Collagen I/III, SMA, Fibrin)

Hematoxylin and Eosin (HE) and Lymphocyte Antigen 6 Complex Locus G6D (1A8-Ly6G) staining was performed with a standardized staining procedure. Collagen fibers were stained using Pico Sirius red (ab150681, Abcam, Cambridge, UK), using polarized light microscopy was used to differentiate collagen I from III. An antibody for smooth muscle actin (SMA, ab5694, Abcam, Cambridge, UK) was applied to the samples, serving as a marker for myofibroblast, which induce wound contraction. Fibrin deposition was determined using a fibrinogen antibody (ab58207, Abcam, Cambridge, UK). Subsequently, the stained samples were incubated according to manufacturer’s instructions. In accordance with each antibody examined, an appropriate isotype control antibody was used as a negative control. All samples were scored semi-quantitatively using following score: (I) None 0: – no signs of tissue staining; (II) Little 1 – small amount of tissue staining; (III) Medium 2 – medium amount of tissue staining; (III) Strong 3 – strong amount of tissue staining. The assessment of collagen alignment was scored based on the orientation of the bundles (0= diffuse with bundles in 90° angle to 4=parallel).

#### Immunofluorescence staining (MPO, NE, citH3, anti-DNA, D1L3, fibrinogen, CCR2, CD68)

3μm-paraffin tissue sections underwent a deparaffinization and rehydration process followed by immunofluorescence staining for myeloperoxidase (MPO), neutrophil elastase (NE), citrullinated histone 3 (citH3), DNA and fibrinogen. Antigen retrieval was assessed by incubating the sample slides with Target Retrieval Solution pH6 (Dako, Santa Clara, USA) in a 97°C water bath for 10 min following a cooling step of 30 min. After rinsing the sections twice for three min with a solution of tri-buffered saline and polysorbate 20 (Tween 20) (TBST), blocking of the probes was performed with a Donkey Block (BioGenex, Fremont, USA) for 30 min at room temperature (RT). Tissue specimens were further incubated with either isotype- or antigen-specific-antibodies at 4°C. Goat anti-mouse MPO (AF 3667, R&D Systems, Minneapolis, USA) diluted 1:20, rabbit anti-mouse NE (AB68672, Abcam, Cambridge, UK) diluted 1:200, rabbit anti-mouse citH3 (AB219406, Abcam, Cambridge, UK) diluted 1:300, rabbit anti-human DNase1L3 (BS7653R, ThermoFisher Scientific, Waltham, MA, USA) diluted 1:100, mouse anti-DNA (CBL186, Merck KGaA Darmstadt, Germany) diluted 1:100, rabbit anti-human fibrinogen (AB92572, Abcam, Cambridge, UK) diluted 1:100, rabbit anti-human CCR2 (PA5-23043, ThermoFisherScientific, Waltham, MA, USA) diluted 1:50, and mouse anti-human CD68 (AB955, Abcam, Cambridge, UK) diluted 1:50 in blocking buffer were used as primary antibody and incubated ON at +4°C. Twelve hours later, sections were rinsed 3x5 min with TBST and subsequently incubated 1:200 with AF647- or Cy5 at RT for 30 min (Jackson ImmunoResearch Europe Ltd., Cambridge, UK). After a 3x 5 min rinsing-step with PBS, nuclei were counterstained by incubating probes with DAPI for 5 min at RT. Finally, slides were rinsed 5 min with H_2_O, and mounted with Fluoromount-G (Southern Biotech, Birmingham, USA). Isotype control antibodies were used as a negative control (MPO = AB-108-C, R&D Systems, Minneapolis, USA; NE = AB37415, citH3 = AB172730, Abcam, Cambridge, UK). Images were taken employing an Aperio VERSA 8 Slide Scanner (Leica Biosystems, Wetzlar, Germany) and processed with Aperio ImageScope Version 12.3.3.5048 (Leica Biosysems). A total of 29 human adhesions were stained (369 sections), on average 13 sections each for the different primary antibodies).

#### Image analyses

Image analyses were performed with Fiji^53^ and with Adobe Photoshop CC Version 19.1.8 (Adobe Inc, Mountain View, CA, USA).

#### Activity of neutrophil elastase

Material from the surgical site of all groups of mice (n=5/group) was washed twice in PBS (ThermoFisher Scientific, 14190250). Then the fluorogenic substrate MeOSuc-AAPV-AMC (Santa Cruz Biotechnology, sc-201163) was added to a final concentration of 100 μM into 48-well plates. Neutrophil elastase from human leukocytes (Sigma-Aldrich, St. Louis, MO, USA, E8140) was used to quantify the activity. To assess the inhibitory effect of the pharmacological and endogenous inhibitors, Sivelestat (final concentration 400 μM, Sigma-Aldrich, S7198) and α1-antitrypsin (final concentration 1 mM, Sigma-Aldrich, A9024) were added to the surgical material, respectively. Fluorescent readings at 37°C were collected on a TECAN Infinite 200 Pro (Tecan, Männedorf, Switzerland) using the filter set (excitation 360nm, emission 465nm) at 20 min intervals. To evaluate the inhibitory potential of Sivelestat and α1-AT we compared the gain of fluorescence two hours before and two hours after addition of the inhibitors.

#### RNA sequencing

All samples were stored in *RNAlater* in 4°C for *24 hours*, and then placed in −80°C freezer until further processing. Sequencing was performed at NovoGene, Peking, China. Treated samples were measured in quadruplets while control samples were measured in triplets and between 36 and 43 M paired-end sequence reads of length 150bp were obtained per replicate. Sequence data have been submitted to the European Nucleotide Archive (ENA). They are publicly available under accession PRJEB40510. Fastp (v0.20.1) was used to remove sequences of sequencing adapters and low quality (Phred quality score below 20) sequences from the 3’-end of the sequence reads.^54^ Thereafter, reads were aligned to the human reference assembly (GRCh38.98) using STAR (v2.7.5c).^55^ Differential expression was assessed with DESeq2.^56^ Genes were considered differentially expressed when the absolute log2FC was 1 or higher and the FDR was 0.1 or lower. WebGestalt (v.2017) was employed for over-representation analysis (ORA) of gene ontology (GO) terms and Reactome pathways.^53^

#### Tryptic digestion of proteins in solution for proteome analyses

The proteins were dissolved in 50 μl lysis buffer consisting of 6 M urea (Sigma, Taufkirchen, Germany), 2 M thiourea (Sigma), 4 % 3-3'-(Cholamidopropyl)-3,3-dimethylammoniumpropylsulfat (CHAPS; Roth, Karlsruhe, Germany), 30 mM dithiothreitol (DTT; Fluka, Seelze, Germany), 2 % IPG-buffer pH 3-10 (GE Healthcare, Freiburg, Germany) using a Bullet Blender® and 0.1 mm glass beads (both from Next Advance, Inc., USA). The protein solution was cleared by centrifugation (20 min @ 12,000 x g, and 10°C) and the protein concentration was determined with the 2D-QuantTM-Kit (GE-Healthcare, Freiburg, Germany). 10 μg of proteins were dissolved in 25 μl ammonium bicarbonate (Fluka) containing 0.1 % ProteasMaxTM (Promega). Cysteines were reduced with 5 mM DTT (30 min @ 50°C) and modified with 10 mM iodacetamide (30 min @ 24°C). The reaction was quenched with an excess of cysteine and trypsin was added to a final concentration of 25 ng/μl resulting in a total volume of 100 μl. After an incubation of 16 hrs at 37°C, the digestion was stopped by addition TFA to a final concentration of 1%. The sample was purified using a C18-ZipTip (Millipore), dried under vacuum and finally dissolved in 10 μl of 0.1% TFA.

#### Liquid-chromatography electrospray-ionization mass spectrometry (LC-ESI-MS)

For analysis, 1 μg of the sample was loaded onto a 50 cm μPACTM C18 column (Pharma Fluidics, Gent, Belgium) in 0.1% formic acid (Fluka) at 35°C. Peptides were eluted with a linear gradient of acetonitrile from 3% to 44% over 60 min followed by a wash with 72% acetonitrile at a constant flow rate of 300 nl/min (ThermoScientific™UltiMate™3000RSLCnano) and infused via an Advion TriVersa NanoMate (Advion BioSciences, Inc. New York, USA) into an Orbitrap Eclipse Tribrid mass spectrometer (ThermoScientific). The mass spectrometer was operating in positive-ionization mode with a spray voltage of the NanoMate system set to 1.7 kV and source temperature at 275°C. Using the data-dependent acquisition mode, the instrument performed full MS scans every 3 seconds over a mass range of m/z 375–1500, with the resolution of the Orbitrap set to 120000. The RF lens was set to 30%, auto gain control (AGC) was set to standard with a maximum injection time of 50 ms. In each cycle the most intense ions (charge state 2-7) above a threshold ion count of 50.000 were selected with an isolation window of 1.6 m/z for HCD-fragmentation at normalized collision energy of 30%. Fragment ion spectra were acquired in the linear IT with a scan rate set to rapid and mass range to normal and a maximum injection time of 100 ms. After fragmentation, the selected precursor ions were excluded for 15 s for further fragmentation. Data acquisition and analysis - Data were acquired with Xcalibur 4.3.73.11. (Thermo Fisher Scientific) and analyzed with Proteome Discoverer 2.5.400 (Thermo Fisher Scientific). The Mascot search engine 2.8.1 (Matrix Science) was used to search against an in-house Uniprot human database. A precursor ion mass tolerance of 10 ppm was used, and one missed cleavage was allowed. The fragment ion mass tolerance was set to 0.8 Da for the linear IT MS2 detection. Carbamidomethylation on cysteines was defined as a static modification and the following optional modifications were considered: oxidation of methionine, citrullinylation, acetylation and carbamylation of lysine and phosphorylation of serine, threonine and tyrosine. The FDR for peptide identification was limited to 0.01 by using a decoy database.

### Quantification and statistical analysis

All data were analyzed using SPSS Statistics 26 (IBM, NY, USA) and GraphPad Prism 9 (GraphPad, CA, USA). A pre-power study calculation was performed using G∗Power 3.1. The power was deducted from previous trials regarding inflammation and NET formation.^14^ Differences between groups were calculated using mixed-effect model with Geisser-Greenhouse correction or Kruskal-Wallis test with Dunn’s correction. Data is presented as mean ± standard deviation (SD). The level of significance was set at 0.05.
