# Supplementary material for: Neutrophil extracellular traps and DNases orchestrate formation of peritoneal adhesions
Source: iScience. 2023 Oct 27;26(12):108289. doi: 10.1016/j.isci.2023.108289 (PMC10682263; doi:10.1016/j.isci.2023.108289)
Supplement: Document S1. Figures S1–S10 [file mmc1.pdf]

iScience, Volume *26*

Supplemental information

Neutrophil extracellular traps and DNases orchestrate formation of peritoneal adhesions

Julia Elrod, Annika Heuer, Jasmin Knopf, Janina Schoen, Lavinia Schönfeld, Magdalena Trochimiuk, Carolin Stiel, Birgit Appl, Laia Pagerols Raluy, Ceren Saygi, Leticija Zlatar, Sami Hosari, Dmytro Royzman, Thomas H. Winkler, Günter Lochnit, Moritz Leppkes, Robert Grützmann, Georg Schett, Christian Tomuschat, Konrad Reinshagen, Martin Herrmann, Tobias A. Fuchs, and Michael Boettcher

Supplement 1: Extracellular traps formation peaks at 72 hours after the induction of adhesions.

Time course of extracellular traps formation using Sytox orange staining of the peritoneal injury site. This supplemental Figure is related to Figure 1.

Supplement 2: Experimental design and treatment strategy of the murine models

(**A**) Experimental design of the adhesion model. Mice were subjected to a 1.5cm long peritoneal incision and two 6x0 Vicryl sutures. They were treated according to their group with either active or inactivated DNases. Experiments ended either after 72 hours (maximum of NET formation) or after 21 days (maximum of adhesion formation). Six groups were used for further experiments. In the treatment group mice received either 10 or 1mg/kg bodyweight Dornase alfa and NTR-10, respectively. This group was compared to shams (neither intervention nor treatment), to the control (received inactivated DNase1) and DNase1-knockout or *Dnase1l3*-knockout mice. This supplemental Figure is related to Figures 1-4.

Supplement 3: DNases affect neutrophil activation and NET formation in mice.

(**A,G**) Ly6G reflects the amount of neutrophil granulocytes. The DNases appear not directly to affect this parameter. (**B-D,F-J**) We assessed neutrophil extracellular traps and neutrophil activation by MPO, NE, and citH3 co-staining. The absence of DNASE1L3 (which reportedly is more tissue bound) significantly increased NETs deposition at the site of peritoneal injury. (**J**) Representative images of the triple staining: DNA-MPO-citH3. Data shown as mean±SD. Statistics: Kruskal-Wallis test with Dunn's correction. For more information on antibodies used, please refer to the STAR Methods section.

Supplement 4: Murine adhesions contain neutrophil elastase activity.

(**A**) Surgical sites from DNase1-KO, DNase1l3-KO, treated WT and WT mice show robust activity of neutrophil elastase (NE). (**B**) The NE activity of the surgical sites is resistant to the inhibition by the pharmacological inhibitor sivelestat and (**C**) the endogenous inhibitor α-1 antitrypsin (α1-AT). For further information on proteins/peptides used, please refer to the STAR Methods section.

Supplement 5: The genes associated with NETs and/or neutrophils are differentially expressed after topical treatment with Dornase alfa.

Treatment with Dornase alfa mostly reduced immune response, leucocyte activation, NET formation and nucleosome assembly. (A) Volcano plot of 3718 significantly differentially expressed genes. 2875 of these genes are downregulated in murine peritoneal cavity cells after DNase1-treatment. (B,C) Volcano plots depicting genes associated with the GO terms regulation of leukocyte activation (B) and activation of immune response (C). Both termini are significantly enriched in DNase1-treated mouse peritoneal cavity cells. The top 20 most significant up and downregulated genes are labeled. (D) Volcano plots depicting previously described NET-associated genes (a) and marker genes of neutrophils (b) [27]. Genes that significantly differed in Dornase alfa versus mock-treated mouse peritoneal cavity cells are labeled. This supplemental Figure is related to Figures 3 and 4.

Supplement 6: Human adhesions contain NETs and DNase1l3.

Expression of the typical NET markers, neutrophil elastase (NE, green), citrullinated histone H3 (citH3, green) and myeloperoxidase (MPO, red) was detected in paraffin sections of surgically removed human adhesions (representative images shown). The presence of DNase1l3 (D1l3, green) was also shown in these adhesions. The enlarged details seen in the immunofluorescence staining and in the HE stained sections correspond to Figure 6. The specificity of all stainings was ensured by the negative signal in the isotype controls corresponding to the markers in the panel above the respective control. The size bar represents 50 μm (25 μm for Supplement 5 (3)). For more information on antibodies used, please refer to the STAR Methods section. This supplemental Figure is related to Figure 5.

Supplement 7: Human adhesions contain NET-borne proteins.

Presence of NET-borne proteins in human adhesions was validated by high-resolution mass spectrometry. As indicated the neutrophil proteins that are established to be bound to NETs like NE, MPO, Cathepsin G, S100-A8, and S100-A9 with high confidence. The secretory Proteinase3 was missing. This supplemental Figure is related to Figure 5.

Supplement 8: Human adhesions contain NETs and Fibrin.

The classical NET markers anti-DNA, citrullinated H3 (citH3), and myeloperoxidase (MPO), all depicted in red, were detected by immunofluorescence staining of surgical specimens of human adhesions. Nuclei were stained by DAPI. The expression of the aforementioned NET markers co-localized with the detection of fibrin (red) in these tissue specimens. The specificity of all stainings was ensured by the negative signal in the isotype controls (wo1st) corresponding to the markers in the panel above the control. The size bar represents 500 μm. For more information on antibodies used, please refer to the STAR Methods section.

Supplement 9: The fibrin from human adhesions is post-translationally modified.

Analyses of the peptide sequences from mass spectrometry of human adhesions obtained by surgery also revealed many oxidized (ox, orange box), citrullinated (cit, yellow box), and carbamylated (Carb, grey box) peptides next to unmodified (w/o, blue box) ones in the alpha, beta and gamma chains of human fibrinogen. The thrombin cleavage site in the alpha chain is depicted in red, whereas the major plasmin cleavage sites in both chains are highlighted in green. * samples were fixed and subjected to antigen retrieval before mass spectrometry analyses. No major differences between fixed and unfixed samples were observed in the peptide analysis. This supplemental Figure is related to Figure 6.

Supplement 10: Plasmin(ogen) displays a lower Mascot score than fibrin and α2-antiplasmin.

The abundance of fibrinolytic proteins in human adhesions was compared to fibrin by high-resolution mass spectrometry. Note, plasmin(ogen) displayed a much lower Mascot score than the fibrin chains in all samples. The inhibitory α2-antiplasmin was higher than plasmin(ogen) and the plasmin activators tPA and uPA were absent in all but one sample. This supplement is related to Figure 6.

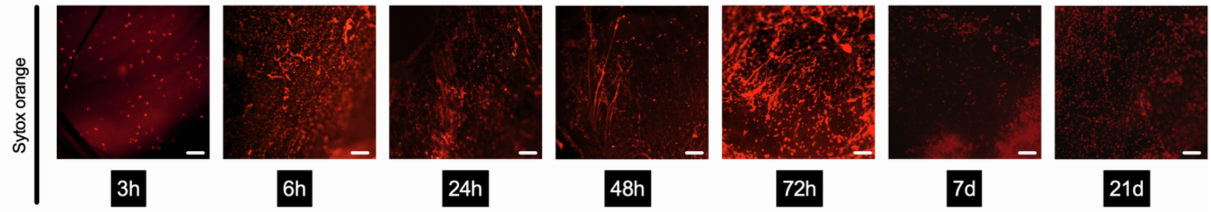

Supplement 1: Time course of extracellular DNA. A: Time course of extracellular traps formation using Sytox orange staining of the peritoneal injury site.

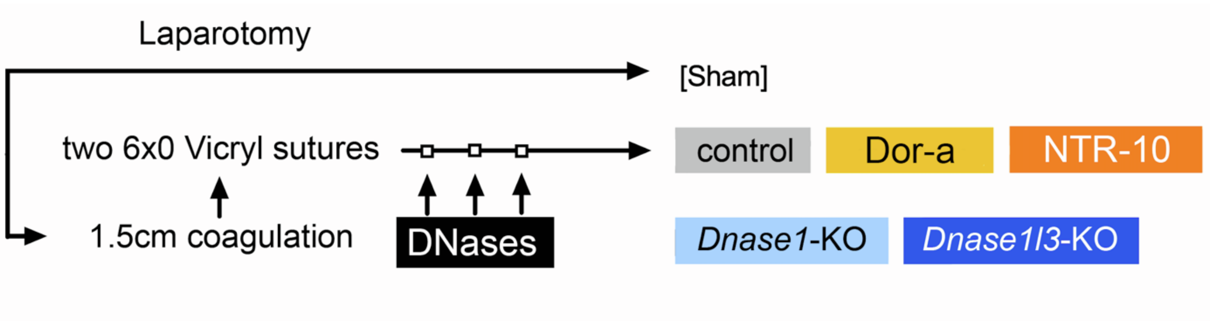

Supplement 2: Experimental design and treatment strategy of the murine models

A: Experimental design of the adhesion model. Mice were subjected to a 1.5cm long peritoneal incision and two 6x0 Vicryl sutures. They were treated according to their group with either active or inactivated DNases. Experiments ended either after 72 hours (maximum of NET formation) or after 21 days (maximum of adhesion formation). Six groups were used for further experiments. In the treatment group mice received either 10 or 1mg/kg bodyweight Dornase alfa and NTR-10, respectively. This group was compared to shams (neither intervention nor treatment), to the control (received inactivated DNase1) and DNase1-knockout or *Dnase1l3*-knockout mice.

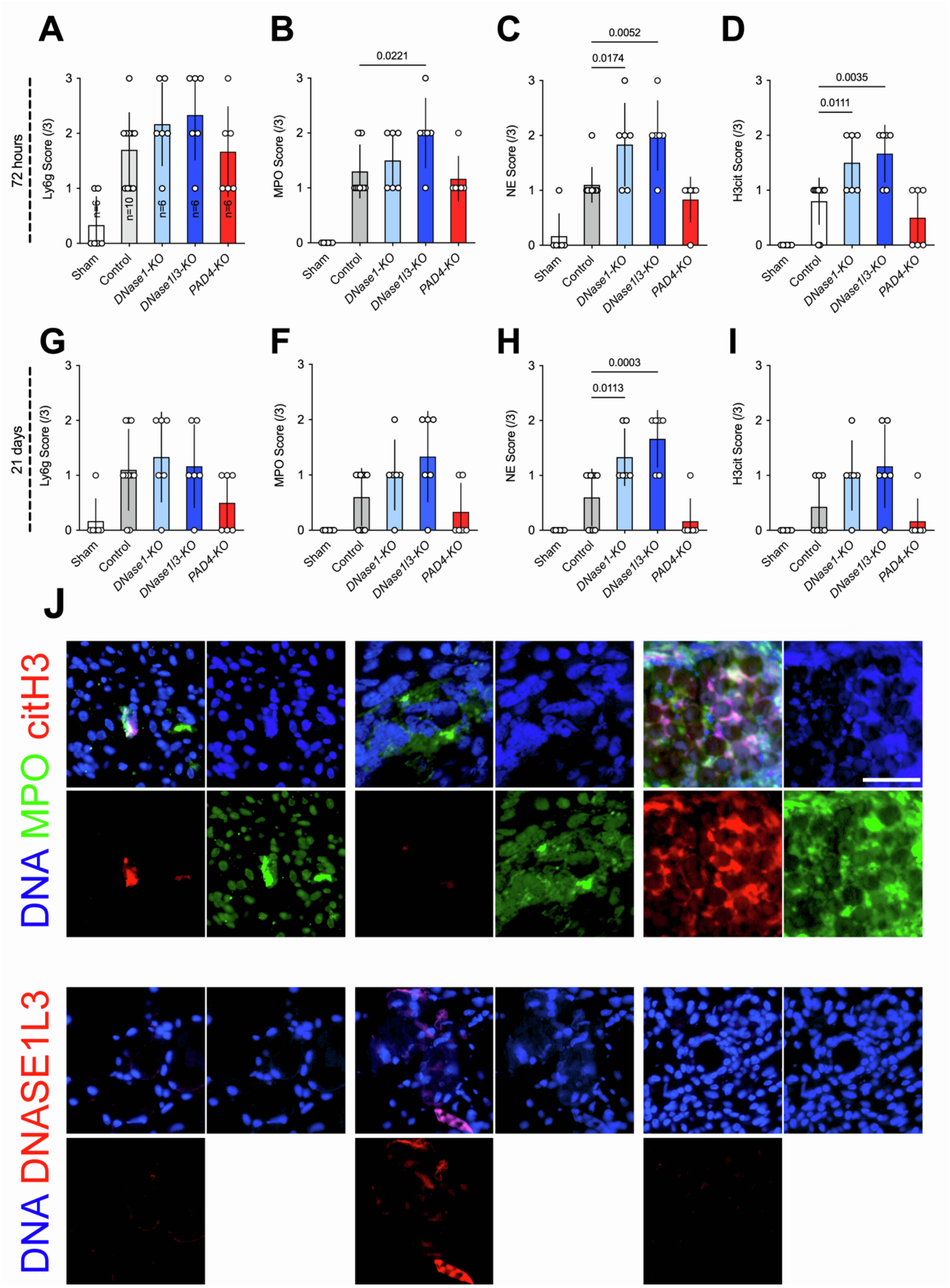

Supplement 3: DNases affect neutrophil activation and NET formation in mice.

(**A,G**) Ly6G reflects the amount of neutrophil granulocytes. The DNases appear not directly to affect this parameter. (**B-D,F-J**) We assessed neutrophil extracellular traps and neutrophil activation by MPO, NE, and citH3 co-staining. The absence of DNASE1L3 (which reportedly is more tissue bound) significantly

increased NETs deposition at the site of peritoneal injury. (**J**) Representative images of the triple staining: DNA-MPO-citH3. Data shown as mean±SD. Statistics: Kruskal-Wallis test with Dunn's correction.

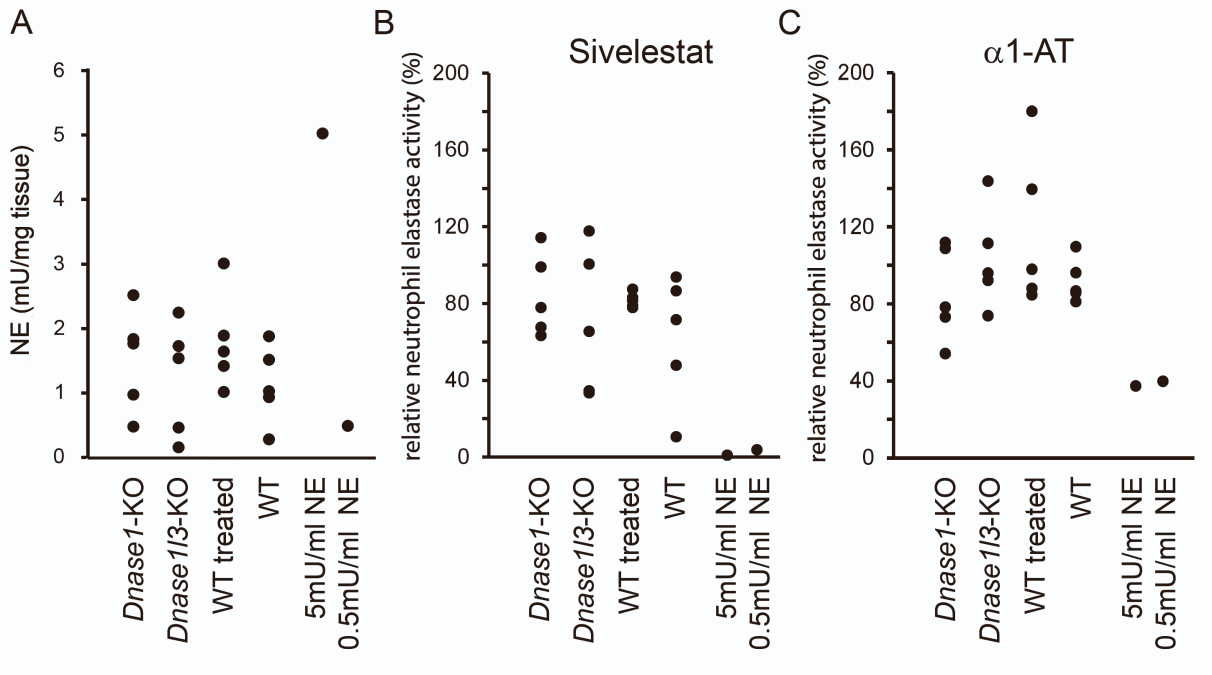

Supplement 4: Murine adhesions contain neutrophil elastase activity.

(**A**) Surgical sites from DNase1-KO, DNase1l3-KO, treated WT and WT mice show robust activity of neutrophil elastase (NE). (**B**) The NE activity of the surgical sites is resistant to the inhibition by the pharmacological inhibitor sivelestat and (**C**) the endogenous inhibitor α-1 antitrypsin (α1-AT).

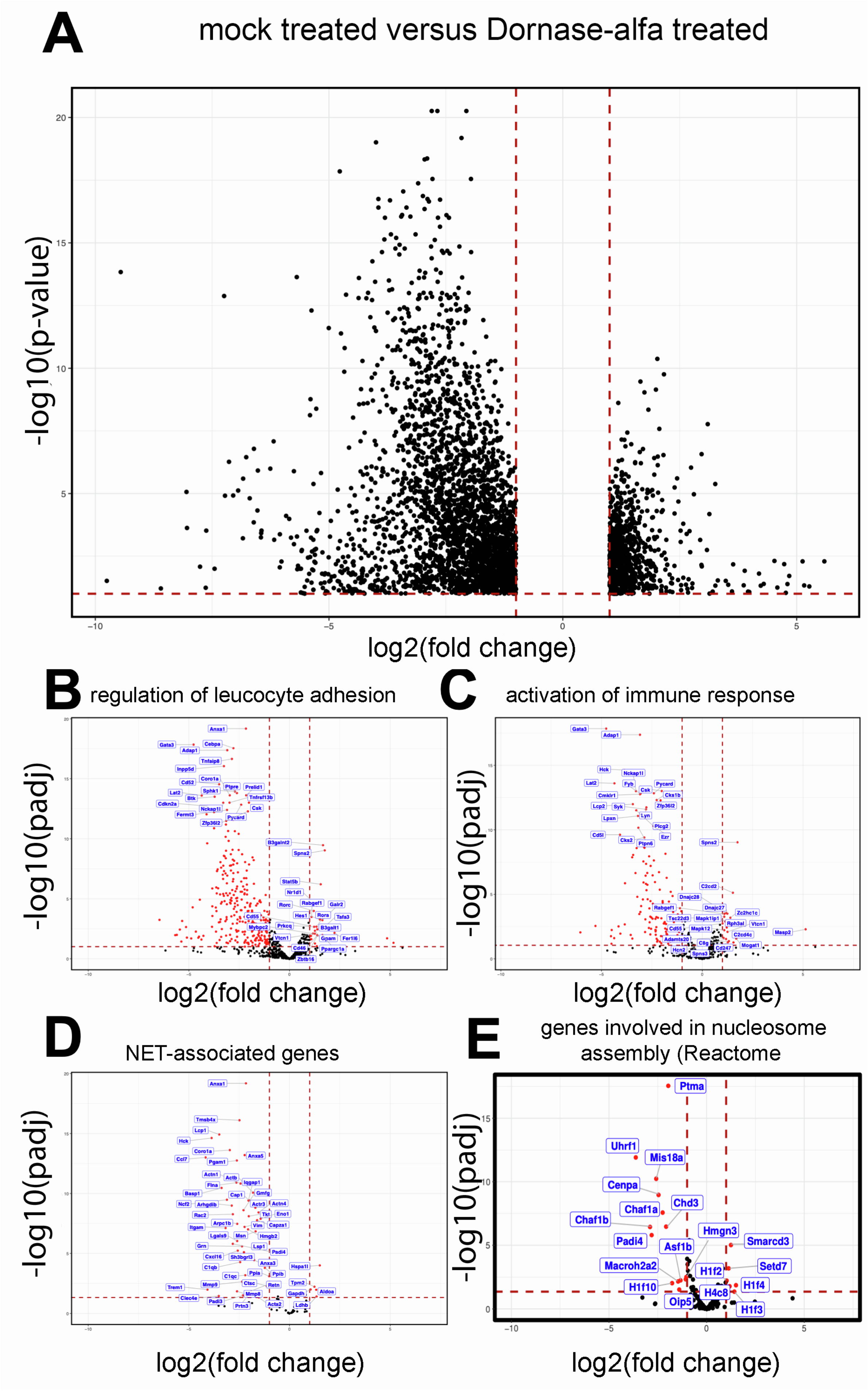
A
mock treated versus Dornase-alfa treated
-log10(p-value)
log2(fold change)
B
regulation of leucocyte adhesion
-log10(padj)
log2(fold change)
C
activation of immune response
-log10(padj)
log2(fold change)
D
NET-associated genes
-log10(padj)
log2(fold change)
E
genes involved in nucleosome
assembly (Reactome
-log10(padj)
log2(fold change)
Ptma
Uhrf1
Mis18a
Cenpa
Chd3
Chaf1a
Chaf1b
Hmgn3
Padi4
Asf1b
Smarcd3
Macroh2a2
H1f2
Setd7
H1f10
H4c8
H1f4
Oip5
H1f3

Supplement 5: The genes associated with NETs and/or neutrophils are differentially expressed after topical treatment with Dornase alfa.

Treatment with Dornase alfa mostly reduced immune response, leucocyte activation, NET formation and nucleosome assembly. (A) Volcano plot of 3718 significantly differentially expressed genes. 2875 of these genes are downregulated in murine peritoneal cavity cells after DNase1-treatment. (B,C) Volcano plots depicting genes associated with the GO terms regulation of leukocyte activation (B) and activation of immune response (C). Both termini are significantly enriched in DNase1-treated mouse peritoneal cavity cells. The top 20 most significant up and downregulated genes are labeled. (D) Volcano plots depicting previously described NET-associated genes (a) and marker genes of neutrophils (b) [27]. Genes that significantly differed in Dornase alfa versus mock-treated mouse peritoneal cavity cells are labeled.

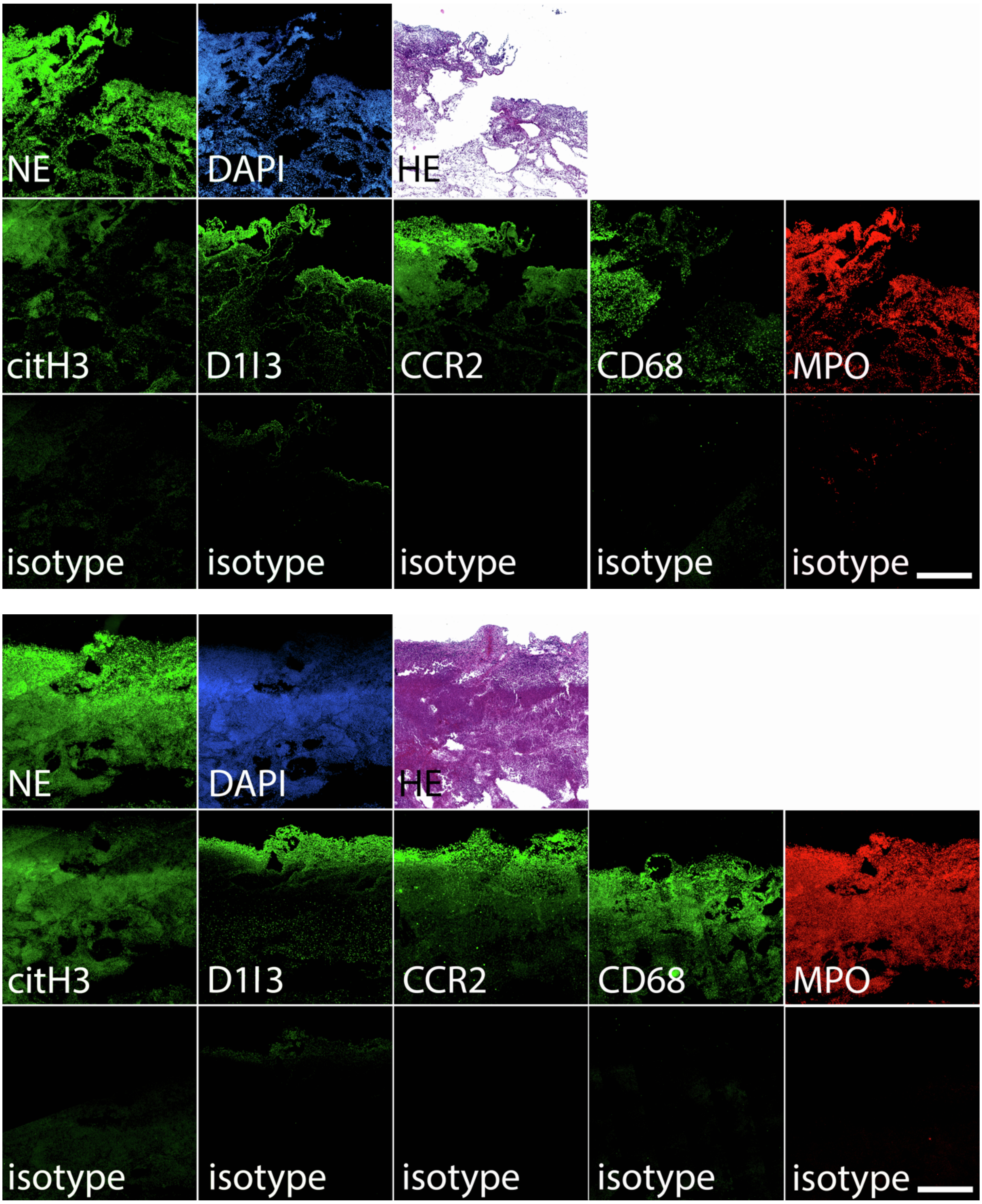

Supplement 6: Human adhesions contain NETs and DNase1l3.

(A,B) Expression of the typical NET markers, neutrophil elastase (NE, green), citrullinated histone H3 (citH3, green) and myeloperoxidase (MPO, red) was detected in paraffin sections of surgically removed human adhesions (representative images shown). The presence of DNase1l3 (D1l3, green) was also shown in these adhesions. The enlarged details seen in the immunofluorescence staining and in the HE stained sections correspond to Figure 6. The specificity of all stainings was ensured by the negative

signal in the isotype controls corresponding to the markers in the panel above the respective control. The size bar represents 50 μm (25 μm for Supplement 5 (3)).

Sample ID	Protein	Coverage [%]	Peptide count	PSMs	Unique Peptides	Amino acid count	MW [kDa]	calc. pI	Score Mascot
A-MB1	NE	42	8	51	8	267	28,5	9,35	1399
A-MB1	MPO	52	35	133	31	745	83,8	8,97	3887
A-MB1	Cathepsin G	50	13	40	13	255	28,8	11,19	1032
A-MB1	S100-A8	94	12	225	12	93	10,8	7,03	4340
A-MB1	S100-A9	96	11	508	11	114	13,2	6,13	7329
A-MB1	Proteinase 3								N.D.
A-MB2	NE	36	6	19	6	267	28,5	9,35	623
A-MB2	MPO	44	29	164	25	745	83,8	8,97	2983
A-MB2	Cathepsin G	36	10	20	10	255	28,8	11,19	268
A-MB2	S100-A8	81	10	97	10	93	10,8	7,03	1779
A-MB2	S100-A9	90	11	290	11	114	13,2	6,13	5427
A-MB2	Proteinase 3								N.D.
A-MB3	NE	44	8	55	8	267	28,5	9,35	1439
A-MB3	MPO	60	42	278	38	777	87,2	9,07	7078
A-MB3	Cathepsin G	51	15	57	15	255	28,8	11,19	1348
A-MB3	S100-A8	77	10	280	10	93	10,8	7,03	4704
A-MB3	S100-A9	93	13	631	13	114	13,2	6,13	8321
A-MB3	Proteinase 3								N.D.
A-MB4	NE								N.D.
A-MB4	MPO	17	6	7	6	745	83,8	8,97	27
A-MB4	Cathepsin G	56	13	29	13	255	28,8	11,19	580
A-MB4	S100-A8	32	3	4	3	93	10,8	7,03	117
A-MB4	S100-A9	85	8	20	8	114	13,2	6,13	321
A-MB4	Proteinase 3								N.D.
A-SH1	NE	42	9	43	9	267	28,5	9,35	847
A-SH1	MPO	50	43	279	38	745	83,8	8,97	5335
A-SH1	Cathepsin G	60	17	97	17	255	28,8	11,19	1802
A-SH1	S100-A8	100	21	583	21	93	10,8	7,03	8730
A-SH1	S100-A9	86	14	667	14	114	13,2	6,13	11315
A-SH1	Proteinase 3								N.D.
A-SH2	NE	54	7	10	7	267	28,5	9,35	203
A-SH2	MPO	49	34	56	30	745	83,8	8,97	745

A-SH2	Cathepsin G	45	9	18	9	255	28,8	11,19	160
A-SH2	S100-A8	59	8	12	8	93	10,8	7,03	82
A-SH2	S100-A9	55	4	11	4	114	13,2	6,13	266
A-SH2	Proteinase 3								N.D.
A-SH3	NE	30	5	16	5	267	28,5	9,35	429
A-SH3	MPO	36	25	110	22	745	83,8	8,97	2210
A-SH3	Cathepsin G	38	10	30	10	255	28,8	11,19	538
A-SH3	S100-A8	72	8	85	8	93	10,8	7,03	1323
A-SH3	S100-A9	82	9	307	9	114	13,2	6,13	3606
A-SH3	Proteinase 3								N.D.
A-SH4	NE	60	11	76	11	267	28,5	9,35	1436
A-SH4	MPO	56	39	310	34	745	83,8	8,97	6791
A-SH4	Cathepsin G	62	17	110	17	255	28,8	11,19	2243
A-SH4	S100-A8	86	12	218	12	93	10,8	7,03	4080
A-SH4	S100-A9	86	12	276	12	114	13,2	6,13	6130
A-SH4	Proteinase 3								N.D.
A-SH5	NE	41	7	89	7	267	28,5	9,35	2393
A-SH5	MPO	45	33	419	26	745	83,8	8,97	9810
A-SH5	Cathepsin G	50	14	105	14	255	28,8	11,19	2415
A-SH5	S100-A8	85	11	1094	11	93	10,8	7,03	13568
A-SH5	S100-A9	91	15	2754	15	114	13,2	6,13	44582
A-SH5	Proteinase 3								N.D.
A-SH6	NE	49	10	288	10	267	28,5	9,35	5140
A-SH6	MPO	66	54	3234	42	745	83,8	8,97	48338
A-SH6	Cathepsin G	60	18	333	18	255	28,8	11,19	5367
A-SH6	S100-A8	8	5	5	1	742	80,2	6,77	43
A-SH6	S100-A9	100	19	1548	19	93	10,8	7,03	25451
A-SH6	Proteinase 3								N.D.
A-SH7	NE	30	5	13	5	267	28,5	9,35	116
A-SH7	MPO	34	24	78	21	745	83,8	8,97	1640
A-SH7	Cathepsin G	40	11	26	11	255	28,8	11,19	416
A-SH7	S100-A8	68	7	35	7	93	10,8	7,03	662
A-SH7	S100-A9	94	10	76	10	114	13,2	6,13	1364
A-SH7	Proteinase 3								N.D.
A-SH8	NE	30	6	35	6	267	28,5	9,35	779
A-SH8	MPO	45	34	167	30	745	83,8	8,97	4122
A-SH8	Cathepsin G	47	12	48	12	255	28,8	11,19	922
A-SH8	S100-A8	70	10	41	10	93	10,8	7,03	776
A-SH8	S100-A9	96	11	98	1	114	13,2	6,13	1900
A-SH8	Proteinase 3								N.D.

Supplement 7: Human adhesions contain NET-borne proteins.

Presence of NET-borne proteins in human adhesions was validated by high-resolution mass spectrometry. As indicated the neutrophil proteins that are established to be bound to NETs like NE, MPO, Cathepsin G, S100-A8, and S100-A9 with high confidence. The secretory Proteinase3 was missing.

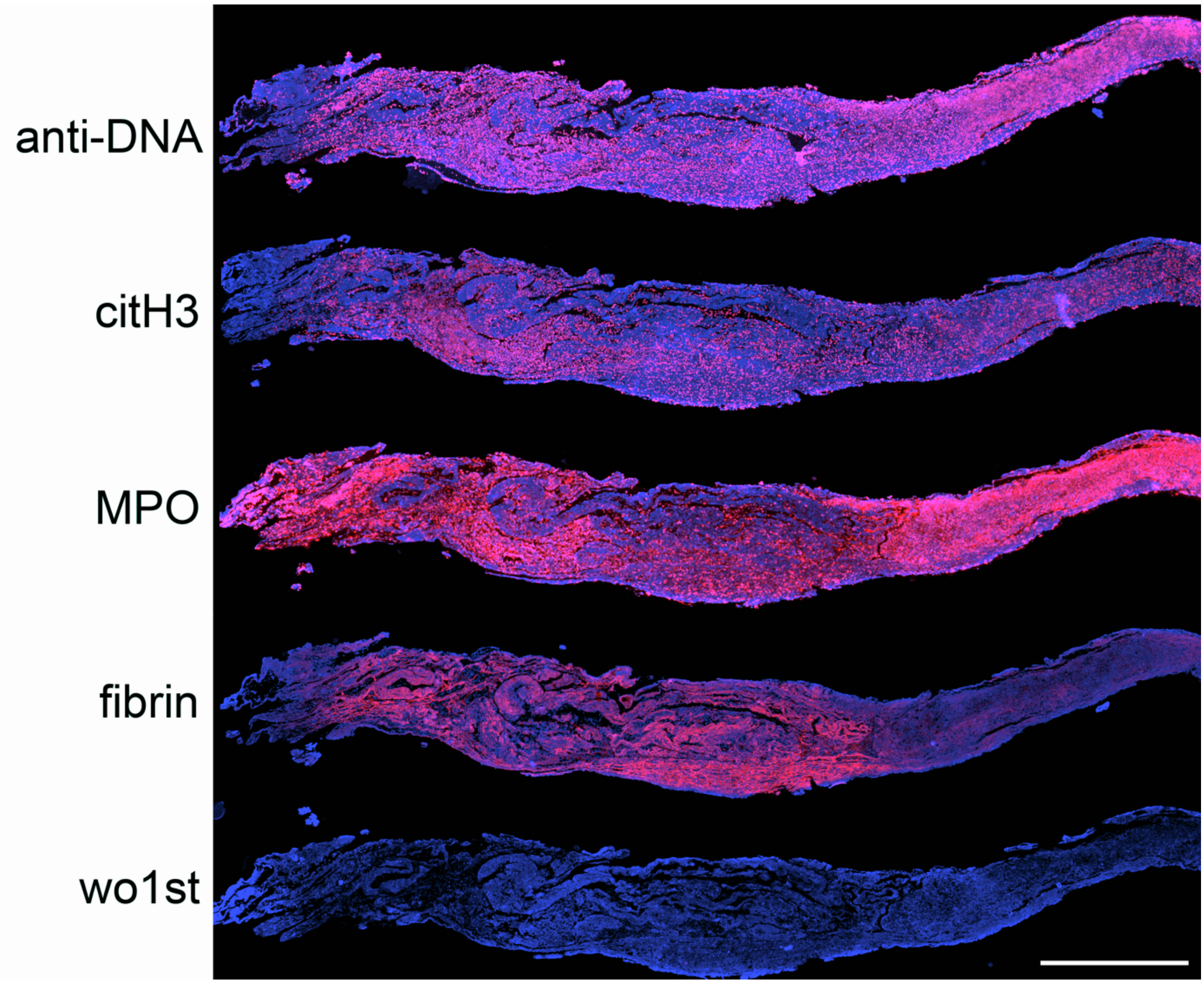

Supplement 8: Human adhesions contain NETs and Fibrin.

(A) The classical NET markers anti-DNA, citrullinated H3 (citH3), and myeloperoxidase (MPO), all depicted in red, were detected by immunofluorescence staining of surgical specimens of human adhesions. Nuclei were stained by DAPI. The expression of the aforementioned NET markers co-localized with the detection of fibrin (red) in these tissue specimens. The specificity of all stainings was ensured by the negative signal in the isotype controls (wo1st) corresponding to the markers in the panel above the control. The size bar represents 500 μm.

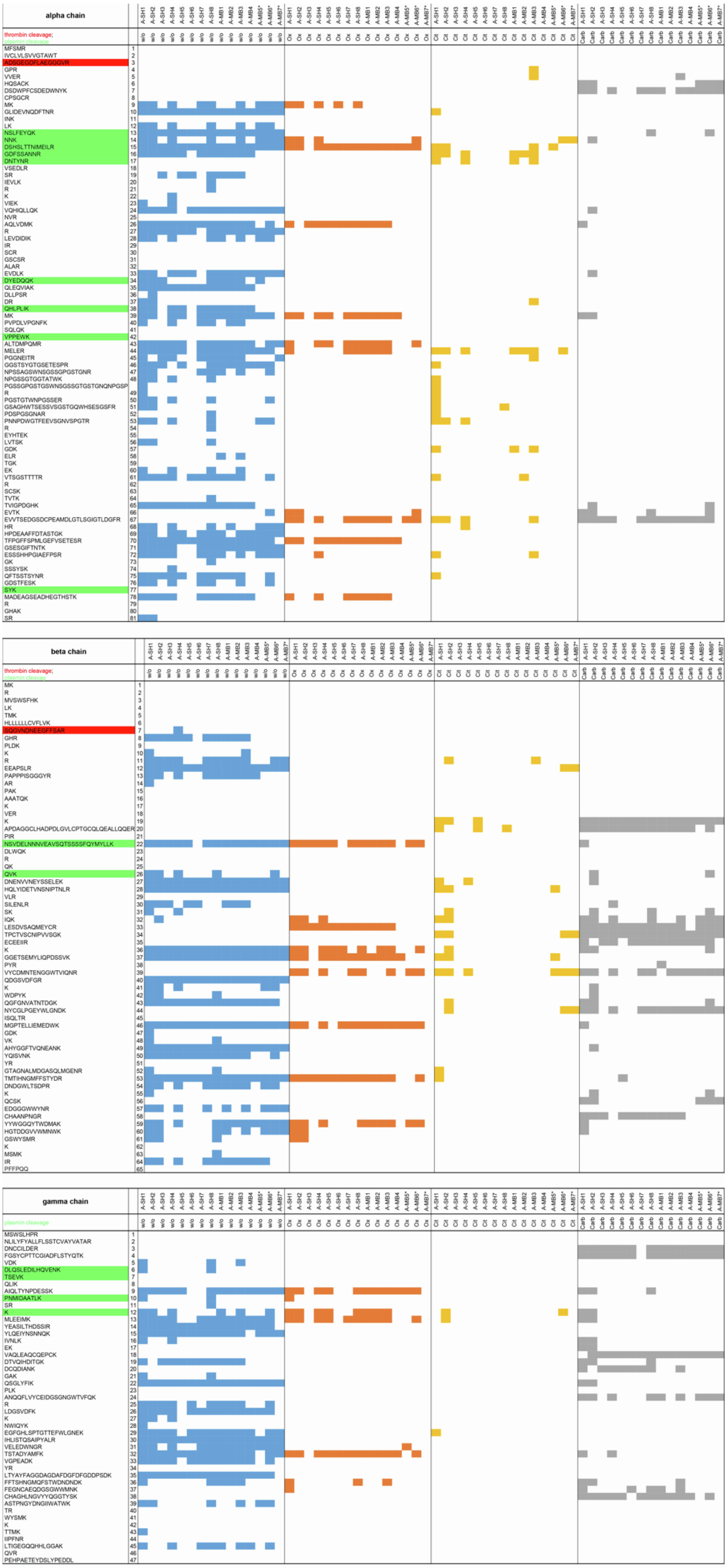
alpha chain
thrombin cleavage;
beta chain
thrombin cleavage;
gamma chain
plasmin cleavage

Supplement 9: The fibrin from human adhesions is post-translationally modified.

(A) Analyses of the peptide sequences from mass spectrometry of human adhesions obtained by surgery also revealed many oxidized (ox, orange box), citrullinated (cit, yellow box), and carbamylated (Carb, grey box) peptides next to unmodified (w/o, blue box) ones in the alpha, beta and gamma chains of human fibrinogen. The thrombin cleavage site in the alpha chain is depicted in red, whereas the major plasmin cleavage sites in both chains are highlighted in green. Samples were fixed and subjected to antigen retrieval before mass spectrometry analyses. No major differences between fixed and unfixed samples were observed in the peptide analysis.

Sample ID	Protein	Coverage [%]	Peptide count	PSMs	Unique Peptides	Amino acid count	MW [kDa]	calc. pI	Score Mascot
A-MB1	Fibrinogen a	51	41	242	41	866	94,9	6,01	5380
A-MB1	Fibrinogen b	77	39	315	39	491	55,9	8,27	9678
A-MB1	Fibrinogen g	76	32	199	6	453	51,5	5,62	5542
A-MB1	a2AP	49	18	42	18	491	54,5	6,29	1452
A-MB1	Plasminogen	67	40	98	30	810	90,5	7,24	2014
A-MB1	uPA-R	21	2	2	2	185	20,7	6,6	94
A-MB1	PAI1								N.D.
A-MB1	PAI2								N.D.
A-MB1	tPA								N.D.
A-MB1	uPA								N.D.
A-MB2	Fibrinogen a	49	42	166	25	866	94,9	6,01	3743
A-MB2	Fibrinogen b	75	45	224	45	491	55,9	8,27	6536
A-MB2	Fibrinogen g	62	25	115	4	445	50,3	6,09	3397
A-MB2	a2AP	27	11	20	11	491	54,5	6,29	559
A-MB2	Plasminogen	30	18	22	14	810	90,5	7,24	324
A-MB2	uPA-R	32	6	6	1	335	37	6,65	112
A-MB2	PAI1								N.D.
A-MB2	PAI2	47	13	18	7	415	46,6	5,63	398
A-MB2	tPA								N.D.
A-MB2	uPA								N.D.
A-MB3	Fibrinogen a	56	60	687	36	866	94,9	6,01	14030
A-MB3	Fibrinogen b	79	49	683	49	491	55,9	8,27	22196
A-MB3	Fibrinogen g	80	35	506	5	453	51,5	5,62	12472
A-MB3	a2AP	42	18	65	18	491	54,5	6,29	1784
A-MB3	Plasminogen	67	42	128	20	810	90,5	7,24	3040
A-MB3	uPA-R	22	5	5	5	335	37	6,65	196
A-MB3	PAI1								N.D.
A-MB3	PAI2								N.D.
A-MB3	tPA								N.D.
A-MB3	uPA								N.D.
A-MB4	Fibrinogen a	33	25	69	25	866	94,9	6,01	1198
A-MB4	Fibrinogen b	63	27	61	27	491	55,9	8,27	1815
A-MB4	Fibrinogen g	57	22	58	22	445	50,3	6,09	1642
A-MB4	a2AP	24	8	11	8	491	54,5	6,29	337
A-MB4	Plasminogen	14	6	7	3	810	90,5	7,24	184

A-MB4	uPA-R								N.D.
A-MB4	PAI1								N.D.
A-MB4	PAI2								N.D.
A-MB4	tPA	7	3	4	3	562	62,9	7,8	21
A-MB4	uPA								N.D.
A-SH1	Fibrinogen a	75	73	1620	62	866	94,9	6,01	23101
A-SH1	Fibrinogen b	88	67	5998	24	491	55,9	8,27	124931
A-SH1	Fibrinogen g	91	56	2939	9	453	51,5	5,62	55164
A-SH1	a2AP	50	23	175	23	491	54,5	6,29	3999
A-SH1	Plasminogen	77	55	155	43	810	90,5	7,24	2229
A-SH1	uPA-R	19	5	5	5	335	37	6,65	78
A-SH1	PAI1	35	11	13	11	402	45	7,2	136
A-SH1	PAI2	22	7	9	1	415	46,6	5,63	190
A-SH1	tPA								N.D.
A-SH1	uPA								N.D.
A-SH2	Fibrinogen a	62	67	794	55	866	94,9	6,01	12072
A-SH2	Fibrinogen b	81	56	1082	3	491	55,9	8,27	21469
A-SH2	Fibrinogen g	79	38	790	4	453	51,5	5,62	13301
A-SH2	a2AP	36	12	40	12	491	54,5	6,29	1201
A-SH2	Plasminogen	67	40	85	34	810	90,5	7,24	1090
A-SH2	uPA-R								N.D.
A-SH2	PAI1								N.D.
A-SH2	PAI2	16	6	6	1	415	46,6	5,63	35
A-SH2	tPA								N.D.
A-SH2	uPA								N.D.
A-SH3	Fibrinogen a	31	26	65	15	866	94,9	6,01	1163
A-SH3	Fibrinogen b	60	30	96	30	491	55,9	8,27	2546
A-SH3	Fibrinogen g	57	24	53	3	453	51,5	5,62	1515
A-SH3	a2AP	23	10	10	10	491	54,5	6,29	177
A-SH3	Plasminogen	23	13	15	10	810	90,5	7,24	228
A-SH3	uPA-R								N.D.
A-SH3	PAI1								N.D.
A-SH3	PAI2								N.D.
A-SH3	tPA								N.D.
A-SH3	uPA								N.D.
A-SH4	Fibrinogen a	57	55	1099	55	866	94,9	6,01	25794
A-SH4	Fibrinogen b	79	48	823	48	491	55,9	8,27	24538
A-SH4	Fibrinogen g	67	35	545	6	453	51,5	5,62	13763
A-SH4	a2AP	62	23	147	23	491	54,5	6,29	3448
A-SH4	Plasminogen	81	55	299	41	810	90,5	7,24	6871
A-SH4	uPA-R								N.D.

A-SH4	PAI1	12	3	3	1	402	45	7,2	73
A-SH4	PAI2	22	7	9	5	415	46,6	5,63	139
A-SH4	tPA								N.D.
A-SH4	uPA								N.D.
A-SH5	Fibrinogen a	27	24	102	24	866	94,9	6,01	1621
A-SH5	Fibrinogen b	60	31	125	31	491	55,9	8,27	3299
A-SH5	Fibrinogen g	54	24	118	3	453	51,5	5,62	2978
A-SH5	a2AP	26	9	14	9	491	54,5	6,29	369
A-SH5	Plasminogen	32	20	27	17	810	90,5	7,24	279
A-SH5	uPA-R	18	5	6	2	335	37	6,65	92
A-SH5	PAI1								N.D.
A-SH5	PAI2								N.D.
A-SH5	tPA								N.D.
A-SH5	uPA								N.D.
A-SH6	Fibrinogen a	21	20	74	20	866	94,9	6,01	1523
A-SH6	Fibrinogen b	59	29	193	29	491	55,9	8,27	5537
A-SH6	Fibrinogen g	57	24	234	4	445	50,3	6,09	6032
A-SH6	a2AP	27	10	31	10	491	54,5	6,29	1052
A-SH6	Plasminogen	30	21	33	17	810	90,5	7,24	725
A-SH6	uPA-R	12	3	3	3	335	37	6,65	55
A-SH6	PAI1	24	8	10	1	402	45	7,2	247
A-SH6	PAI2								N.D.
A-SH6	tPA								N.D.
A-SH6	uPA								N.D.
A-SH7	Fibrinogen a	42	35	179	20	866	94,9	6,01	3926
A-SH7	Fibrinogen b	74	38	191	38	491	55,9	8,27	4375
A-SH7	Fibrinogen g	66	27	136	8	445	50,3	6,09	2875
A-SH7	a2AP	25	11	13	11	491	54,5	6,29	152
A-SH7	Plasminogen	21	13	20	13	810	90,5	7,24	291
A-SH7	uPA-R								N.D.
A-SH7	PAI1								N.D.
A-SH7	PAI2	5	2	2	1	415	46,6	5,63	38
A-SH7	tPA								N.D.
A-SH7	uPA								N.D.
A-SH8	Fibrinogen a	64	73	878	43	866	94,9	6,01	16739
A-SH8	Fibrinogen b	86	59	667	59	491	55,9	8,27	12687
A-SH8	Fibrinogen g	84	48	499	9	453	51,5	5,62	9521
A-SH8	a2AP	38	16	41	16	491	54,5	6,29	1120
A-SH8	Plasminogen	54	35	73	28	810	90,5	7,24	1485
A-SH8	uPA-R								N.D.
A-SH8	PAI1								N.D.

A-SH8	PAI2	N.D.
A-SH8	tPA	N.D.
A-SH8	uPA	N.D.

Supplement 10: The plasminogen activators are virtually absent at adhesion site.

The abundance of fibrinolytic proteins in human adhesions was compared to fibrin by high-resolution mass spectrometry. Note, plasmin(ogen) displayed a much lower Mascot score than the fibrin chains in all samples. The inhibitory α2-antiplasmin was higher than plasmin(ogen) and the plasmin activators tPA and uPA were absent in all but one sample.
